# Wavelet-Based Genomic Signal Processing for Centromere Identification and Hypothesis Generation

**DOI:** 10.3389/fgene.2019.00487

**Published:** 2019-05-31

**Authors:** Deborah Weighill, David Macaya-Sanz, Stephen Paul DiFazio, Wayne Joubert, Manesh Shah, Jeremy Schmutz, Avinash Sreedasyam, Gerald Tuskan, Daniel Jacobson

**Affiliations:** ^1^The Bredesen Center for Interdisciplinary Research and Graduate Education, University of Tennessee, Knoxville, TN, United States; ^2^Biosciences Division, Oak Ridge National Laboratory, Oak Ridge, TN, United States; ^3^Department of Biology, West Virginia University, Morgantown, WV, United States; ^4^Oak Ridge Leadership Computing Facility, Oak Ridge National Laboratory, Oak Ridge, TN, United States; ^5^Department of Energy Joint Genome Institute, Walnut Creek, CA, United States; ^6^HudsonAlpha Institute for Biotechnology, Huntsville, AL, United States

**Keywords:** *Populus trichocarpa* centromeres, wavelet transform, DNA methylation, SNP density, CENH3, co-evolution, data integration

## Abstract

Various ‘omics data types have been generated for *Populus trichocarpa*, each providing a layer of information which can be represented as a density signal across a chromosome. We make use of genome sequence data, variants data across a population as well as methylation data across 10 different tissues, combined with wavelet-based signal processing to perform a comprehensive analysis of the signature of the centromere in these different data signals, and successfully identify putative centromeric regions in *P. trichocarpa* from these signals. Furthermore, using SNP (single nucleotide polymorphism) correlations across a natural population of *P. trichocarpa*, we find evidence for the co-evolution of the centromeric histone CENH3 with the sequence of the newly identified centromeric regions, and identify a new CENH3 candidate in *P. trichocarpa*.

## 1. Introduction

Integrating data from multiple different sources is a task which is becoming more prevalent with the increased availability of systems biology data from high-throughput ‘omics technologies and phenotyping strategies (Gomez-Cabrero et al., 2014). Developing statistical and mathematical approaches to integrate this data in order to provide an increased understanding of the biological system is thus an important endeavor. For the bioenergy feedstock crop *Populus trichocarpa*, several heterogenous datasets have been generated. The full genome sequence is available and is currently in its third version (Tuskan et al., 2006). A large collection of ~28,000,000 Single Nucleotide Polymorphisms (SNPs) called across 882 genotypes are publicly available (https://doi.ccs.ornl.434gov/ui/doi/55), which were derived from the resequenced genomes of ~1,000 *P. trichocarpa* genotypes propagated in common gardens (Tuskan et al., 2011; Slavov et al., 2012; Evans et al., 2014). Methyl-DNA immunoprecipitation (MEDIP)-seq DNA methylation data is also available for 10 different *P. trichocarpa* tissues (Vining et al., 2012). A gene expression atlas for *P. trichocarpa* is also available on Phytozome (Goodstein et al., 2012).

Integration of multiple heterogeneous data types requires coercing them into mathematical structures that allow them to be compared/merged/layered. For example, each of the data types mentioned above provides feature(s) which can be represented as vectors of numbers, with each vector representing a signal which varies across a chromosome, for example, the gene density across a chromosome, or the methylation profile of a chromosome. Once represented as a signal, these data types are amenable to signal processing techniques. This study aims to make use of signal processing techniques of these multiple data types in order to attempt to identify chromosome structural features in *P. trichocarpa*.

The centromere is an important chromosomal structure which controls the segregation of chromosomes during cell division, and is the location for the assembly for the kinetochore protein complex (O'Connor, 2008; Feng et al., 2015). Centromeric chromatin contains a histone H3 variant specific to the centromere (CENH3), which has been found in many organisms, including plants (Talbert et al., 2002). Studies by Henikoff et al. (2001) and Cooper and Henikoff (2004) have suggested that CENH3 is co-evolving with the sequence of the centromere.

Centromeric regions can vary in size, and can be small regions consisting of only one nucleosome, such as in *Saccharomyces cerevisiae* (Furuyama and Biggins, 2007; Feng et al., 2015), while plant centromeric regions are large (Mb scale), and consist of repetitive sequences (Mehrotra and Goyal, 2014; Feng et al., 2015). Centromeres also have epigenetic characteristics in that plant centromeric regions have been found to be relatively highly methylated (Zhang et al., 2006; Vining et al., 2012).

Previously, putative centromere positions were identified in *P. trichocarpa* as chromosomal regions of low gene density and high methylation, presented visually, but coordinates were not reported (Vining et al., 2012). Putative centromere positions have also been identified based on recombination rates along chromosomes through visual inspection of profiles of 4*N*_*e*_*c* (Slavov et al., 2012). Cossu et al. (2012) identified putative centromeric repeats of *P. trichocarpa* which identified putative centromere positions on some of the *P. trichocarpa* chromosomes in a previous assembly of the genome. In Pinosio et al. (2016), putative centromeres were identified as regions as the 250 kb window on each chromosome with the lowest gene density, and reported enrichment of insertions and repetitive elements in the centromeric regions. However, to our knowledge, there has not been a comprehensive study of *P. trichocarpa* centromeres integrating various available data types and multiple lines of evidence.

The large collection of data available for *P. trichocarpa* provides a source of multiple features which can be represented as density signals across each chromosome. Certain features, such as gene density and SNP density, can be readily constructed from the data available. Other lines of evidence, such as SNP correlation/co-segregation need to be calculated from the data before the chromosome signals can be constructed.

**Figure 1:**
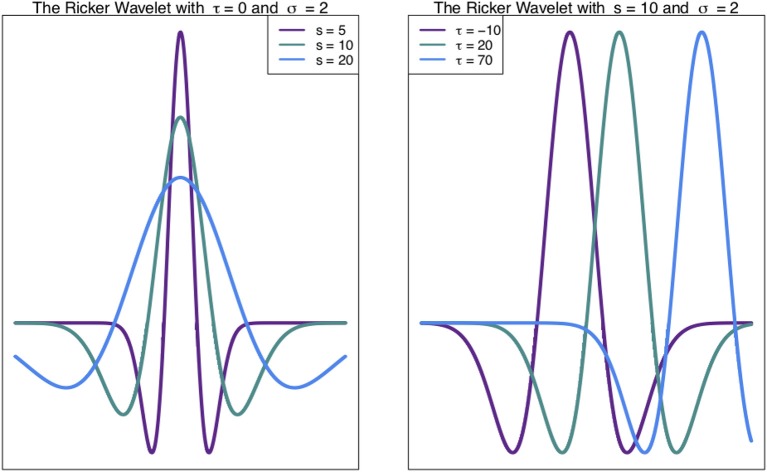
Ricker Wavelet. The Ricker wavelet shown for different values of scale *s* and translation τ in Equation (1) (Leavey et al., 2003; Machado et al., 2011).

Such chromosome signals contain variation on multiple scales, including high frequency (narrow) peaks and low-frequency (broad) peaks. These different scales of peaks contain different information. Thus, techniques to analyse these signals at different scales are valuable (see Spencer et al., 2006; McCormick et al., 2017). The Wavelet Transform, a signal processing technique, can be used to unpack the information in different scales of a signal, such as a density profile across a chromosome (Spencer et al., 2006). In general, the wavelet transform involves expressing a function (signal) as a linear combination of functions called wavelets. These functions are scaled translations of a mother wavelet, such as the Ricker Wavelet (Figure 1). What results from a wavelet transform is a wavelet coefficient *W*(*s*, τ) (Equation 1), for every scale *s* and translation (shift along the x-axis) τ (Leavey et al., 2003).

(1) $$\begin{array}{r} W\left(s,\tau \right)=\frac{1}{\sqrt{s}}\int f\left(t\right)\psi^{*}\left(\frac{t-\tau }{s}\right)dt \end{array}$$

Given the peak-like shape of the wavelet, a wavelet coefficient will indicate “how much of a peak” is present at a particular scale and at a particular position of the signal. Thus, the wavelet transform allows us to investigate the peaks of a signal at different scales and locations.

This study makes use of the Continuous Wavelet Transform (CWT) in characterizing chromosomal gene density, SNP density and methylation density signals in *P. trichocarpa*. We use the resulting CWT coefficient landscapes to identify the putative centromere locations and illustrate the wavelet signature of a centromere. We also investigate potential co-evolution signatures between the centromeric histone CENH3 and the newly identified centromeric regions through the calculation of SNP correlations across the population, and find evidence supporting the hypothesis of the co-evolution of putative *P. trichocarpa* CENH3 genes with the centromere sequences in *P. trichocarpa*. While wavelets have previously been used in chromosome classification (Wu and Castleman, 2000), and the discrete wavelet transform has been used in the analysis of feature profiles across a chromosome in human (Spencer et al., 2006), to our knowledge this work presents the first use of the continuous wavelet transform in the identification of centromere positions from SNP and methylation density profiles. This study provides an example of how signal processing of multiple data types can be used to generate hypotheses surrounding the structure of chromosomes.

## 2. Methods and Materials

### 2.1. Variant Data and SNP Correlations

*Populus trichocarpa* (Tuskan et al., 2006) variant data (doi: 10.13139/OLCF/1411410) was obtained from https://doi.ccs.ornl.434gov/ui/doi/55. This dataset consists of SNP 28,342,758 SNPs called across 882 *P. trichocarpa* genotypes and is derived the whole genome resequencing of a Genome Wide Association Study (GWAS) population clonally replicated in common gardens (Tuskan et al., 2011).

The most reliable SNPs within the dataset were selected, consisting of the 90% tranche (the tranche recovering 90% of the “true” SNPs). VCFtools (Danecek et al., 2011) was used to extract the desired Tranche of SNPs from the VCF file and reformat it into .tfam and .tped files. Plink (Purcell et al., 2007, http://pngu.mgh.harvard.edu/purcell/plink/) was used to determine the minor allele frequency (MAF) and the call rate (fraction alleles observed) for each SNP, and removed all SNPs with MAF ≤ 0.01 and call rate ≤ 0.5.

Correlations between all pairs of SNPs were calculated using the Custom Correlation Coefficient (CCC) (Climer et al., 2014a,b). This was performed on both the filtered set of SNPs as well as the entire 90% tranche, using a new, GPU implementation of the CCC metric for the calculation of SNP correlations (Joubert et al., 2017) as well as the original software (Climer et al., 2014a,b), respectively. Calculation of the CCC between all pairs of SNPs using the original software was performed in parallel, as described in Weighill et al. (2018). Briefly, the CCC between allele *x* at location *i* and allele *y* and location *j* is defined as:

(2) $$\begin{array}{r} CCC_{i_{x}j_{y}}=\frac{9}{2}R_{i_{x}j_{y}}\left(1-\frac{1}{f_{i_{x}}}\right)\left(1-\frac{1}{f_{j_{y}}}\right) \end{array}$$

where *R*_*i*_*x*_*j*_*y*__ is the relative co-occurrence of allele *x* at location *i* and allele *y* at location *j*, *f*_*i*_*x*__ is the frequency of allele *x* at location *i* and *f*_*j*_*y*__ is the frequency of allele *y* at location *j*.

This was performed in a parallel fashion by constructing a Perl wrapper around the ccc binary, making use of the Parallel::MPI::Simple Perl module, developed by Alex Gough and available on The Comprehensive Perl Archive Network (CPAN) at www.cpan.org. “The set of ~10 million SNPs was divided into 20 different blocks, and the CCC was calculated for each within-block and cross-block comparison in separate jobs, to a total of 210 MPI jobs …A threshold of 0.7 was then applied.” (Quotation from Weighill et al., 2018).

### 2.2. Chromosome Feature Profile Construction

#### 2.2.1. SNP Density Profiles

A SNP density profile was created for each chromosome using the filtered set of SNPs by counting the number of these SNPs in non-overlapping 10 kb windows across the chromosome.

#### 2.2.2. Methylation Profiles

Methylation (MeDIP-seq) data from 10 *P. trichocarpa* tissues generated from the study by Vining et al. (2012) re-aligned to the version 3 assembly of *P. trichocarpa* was downloaded from Phytozome (Goodstein et al., 2012). This data consists of MeDIP-seq reads from tissues including bud, callus, female catkin, internode explant, leaf, make catkin, phloem, regenerated internode, root and xylem tissue.

Samtools (Li et al., 2009) was used to view the data and BamTools stats (Barnett et al., 2011) was used to investigate statistics of the reads in the bam files. BEDTools (Quinlan, 2014) was used to count the number of reads mapped to 10 kb windows across the genome. This will allow us to construct a “mapped read density” distribution for each tissue and each chromosome, showing the number of reads which mapped to different regions of the genome, and thus indicating methylation hotspots. The BEDOPs (Neph et al., 2012) software was used to convert .gtf files of the 10kb windows per chromosome into .bed files. GNU-Parallel (Tange, 2011) was used to run the BEDTools jobs in parallel.

#### 2.2.3. Gene Density Profiles

Gene density profiles were constructed for each chromosome. Gene density for a given window was defined as the number of nucleotide positions within that window that reside within genes. Gene boundaries were determined from the Ptrichocarpa_210_v3.0.gene.gff3 annotation file obtained from the *P. trichocarpa* version 3 genome annotation (Tuskan et al., 2006) available on Phytozome (Goodstein et al., 2012) through the genome portal of the Department of Energy Joint Genome Institute (Grigoriev et al., 2011; Nordberg et al., 2014).

#### 2.2.4. Genome Gap Density Profiles

Genome gap density profiles were constructed for each chromosome, similar to the approach for constructing SNP density profiles. For each non-overlapping 10kb window on a chromosome, the number of “N” positions were counted in the genome assembly file ptrichocarpa_210_v3.0.fa obtained from the version 3 genome assembly (Tuskan et al., 2006) available on Phytozome (Goodstein et al., 2012).

### 2.3. Continuous Wavelet Transform of Chromosome Feature Profiles

The CWT was performed on chromosome feature density profiles using the wmtsa R wavelet package (Percival and Walden, 2006; Constantine and Percival, 2016), the R programming language (R Core Team, 2015), RStudio (RStudio Team, 2016), and various R packages and resources (Constantine et al., 2016). The CWT results in sets of wavelet coefficients at different scales. These were plotted as a heatmap/coefficient landscape, showing the numerical values of the different wavelet coefficients across the signal, at different scales. Plots were generated using custom R scripts and R packages (Neuwirth, 2014; R Core Team, 2015; Nychka et al., 2017).

### 2.4. Centromere Position Identification

Putative centromeres were located for each chromosome by computationally identifying the “tooth-X-ray” signature in the wavelet landscapes. Let the matrix *M* represent the methylation wavelet landscape and let *S* represent the SNP wavelet landscape for a given chromosome. We identified the maximum wavelet coefficient in the upper third of the methylation wavelet landscape (internode explant tissue), and identified the scale *p* (row of *M*) at which this maximum coefficient was found. This identified the general pericentromeric scale. The borders of the approximate pericentromeric regions *b*_1_ and *b*_2_ were identified as the zeroes of the methylation wavelet coefficient vector at scale *p* (Supplementary Note S1, Figure S1). The minimum wavelet coefficient in the lower two thirds of *S* between the borders *b*_1_ and *b*_2_ was then identified, and the scale *c* (row of *S*) at which this minimum occurs was considered the centromeric scale. The methylation pericentromeric scale vector *M*_*p*_ (row *p* in matrix *M*) and the SNP centromeric scale vector *S*_*c*_ (row *c* of matrix *S*) were extracted, and scaled to have mean 0 and standard deviation 1. The approximate centromere locations were then identified as the position *x* at which the maximum

(3) $$\begin{array}{r} \text{max}\left(M_{p,x}^{*}-S_{c,x}^{*}\right) \end{array}$$

is obtained, where $M_{p,x}^{*}$ and $S_{c,x}^{*}$ represent the *x*th entry in the scaled vectors of *M*_*p*_ and *S*_*c*_, respectively.

See Supplementary Note S1 and Figure S1 for further details.

### 2.5. Centromere Repeat Sequence Profiles

Plant centromere repeat sequences were downloaded from the PGSB Repeat Database (Nussbaumer et al., 2012) at http://pgsb.helmholtz-muenchen.de/plant/recat/index.jsp. The repeat sequences were then BLASTed (Altschul et al., 1990) against the *P. trichocarpa* version 3 genome on Phytozome (Goodstein et al., 2012), using an E-value threshold of 10^−5^ and other default parameters. A density profile of BLAST hits was then constructed for each chromosome. The BLAST hit density for a given 10 kb window was defined as the number of positions within the window that lay within a BLAST hit (*E*-value ≤ 10^−5^) with a plant centromeric repeat sequence. We obtained putative *P. trichocarpa* centromeric repeat sequences from Cossu et al. (2012), and constructed a BLAST hit density profile for these repeat sequences in a similar manner. These centromere repeat density profiles were visualized alongside of the predicted putative centromere positions.

### 2.6. Synteny Analysis

Syntenic blocks within the *P. trichocarpa* version 3.0 genome were constructed using CoGe SynMap (Lyons et al., 2008; Haug-Baltzell et al., 2017). Syntenic segments were computed based on gene order, within a maximum of 10 non-matching genes between matching genes, and a minimum of five aligned genes per segment, similar to the parameters used in the syntenic block analysis of the original genome (Tuskan et al., 2006). Synonymous substitution rates (Ks) were also calculated. Syntenic blocks were visualized using Circos (Krzywinski et al., 2009). For each chromosome, syntenic blocks which overlapped with putative centromere locations on a chromosome were extracted.

### 2.7. Co-expression Network

Gene co-expression relationships were queried on PhytoMine though Phytozome (Goodstein et al., 2012; Kalderimis et al., 2014). A custom co-expression network was also created as described in Weighill et al. (2018) using the *P. trichocarpa* (Nisqually-1) RNA-seq dataset from JGI Plant Gene Atlas project (Sreedasyam et al., unpublished). This dataset consists of samples for standard tissues (leaf, stem, root and bud tissue) and libraries generated from nitrogen source study. A list of sample descriptions was accessed from Phytozome at https://phytozome.jgi.doe.gov/phytomine/aspect.do?name=Expression. Networks were visualized in Cytoscape (Shannon et al., 2003).

### 2.8. Co-evolution of Putative CENH3 Genes

The genomic sequence of the *Arabidopsis thaliana* CENH3 gene (AT1G01370) was obtained from Phytozome (Goodstein et al., 2012) and BLASTed against the *P. trichocarpa* version 3 genome (Tuskan et al., 2006) on Phytozome using default parameters. Two BLAST hits were obtained, one gene on chromosome 14 (Potri.014G096400) and one on chromosome 2 (Potri.002G169000). While Potri.014G096400 contains functional annotations on Phytozome, including Panther PTHR11426:SF46 (“Histone H3-like centromeric protein A”) and Pfam PF00125 (“Core histone H2A/H2B/H3/H4SNPs”), Potri.002G169000 contains no functional annotations, likely because of sequencing/assembly issues. There are various exons predicted in the gene which are not considered to be translated. However, when searching for domains in the genome sequence of Potri.002G169000 using CD-search at NCBI (Marchler-Bauer and Bryant, 2004; Marchler-Bauer et al., 2010, 2014), Pfam PF00125 (“Core histone H2A/H2B/H3/H4SNPs”) is identified in the sequence. Thus, we have two valid CENH3 candidates. SNPs which correlated with SNPs within these genes (CCC ≥0.7) were extracted from the SNP correlations. Density profiles of these SNPs were then constructed for all chromosomes in non-overlapping 10 kb bins, similar to the profile construction described above.

## 3. Results and Discussion

### 3.1. Chromosome Feature Profiles and CWT Coefficient Landscapes

**Figure 2:**
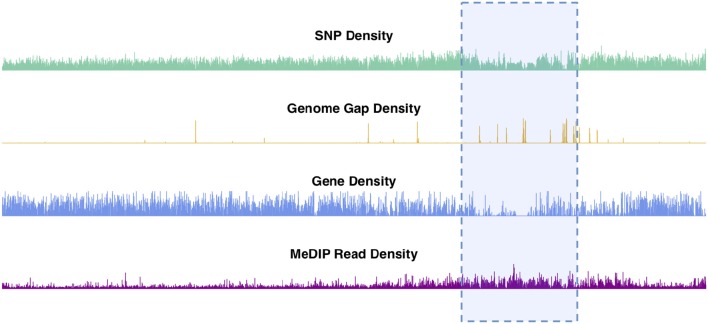
Chromosome 2 feature density signals. Feature density signals for SNP, gene, MeDIP read (internode explant tissue) and genome gap density in 10 kb windows across *P. trichocarpa* chromosome 2.

Chromosomal features including SNPs, genes, genome gaps and DNA methylation plotted as density signals across a chromosome result in signals that vary along the length of the chromosome (Figure 2, Figures S2–S20). These profiles show the frequency of a particular feature in 10kb bins across each chromosome. These profiles vary on different scales, in that they contain peaks and valleys of different frequencies/broadness. Each of these signals has fine variation in the form of narrow, high frequency peaks, as well as broad, low-frequency peaks, as illustrated in the feature density profiles of chromosome 2 (Figure 2). The highlighted region in Figure 2 indicates the most prominent broad-scale feature, consisting of a large-scale valley in the SNP and gene density profiles, and a large-scale peak in the methylation (MeDIP-Seq read density) profile.

**Figure 3:**
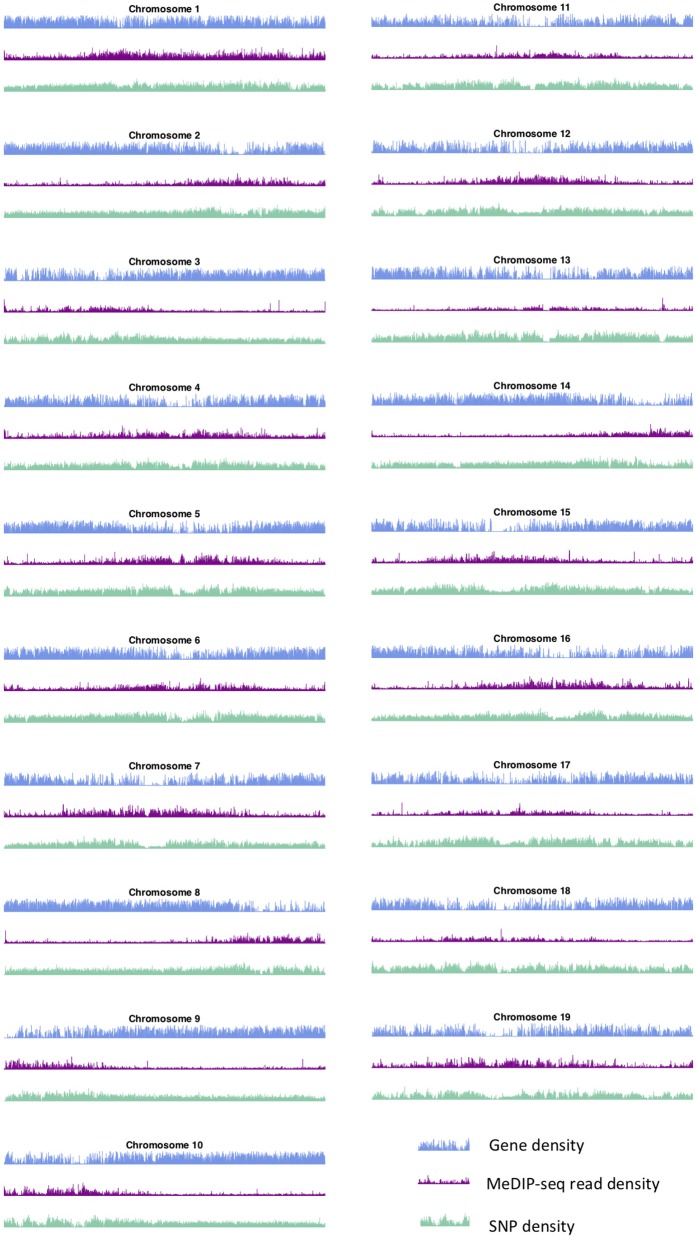
Methylation, SNP, and gene density. SNP, gene, and methylation (internode explant tissue) density profiles for all chromosomes of *P. trichocarpa*.

These large-scale peak-valley combinations of SNP, gene and methylation density profiles are observed easily on all chromosomes (Figure 3). One can see a large-scale peak in the methylation profile coinciding with valleys in the gene density and SNP density signals on each chromosome. The locations of these large-scale peak-valley combinations seem to agree with the putative *P. trichocarpa* centromere positions proposed by Vining et al. (2012) on the basis of high methylation read coverage, high repeat-to-gene ratios and recombination valleys, and also agrees with some of the putative centromere positions identified through repeat elements (Cossu et al., 2012).

**Figure 4:**
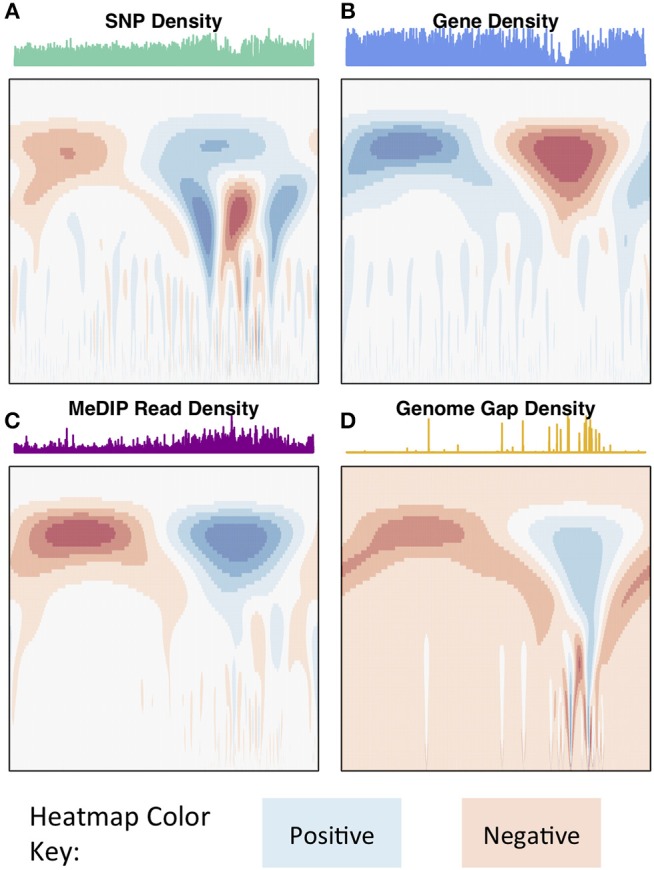
Chromosome 2 CWT landscapes. CWT Coefficient landscapes of chromosome 2 for **(A)** SNP density, **(B)** gene density, **(C)** methylation (MeDIP-Seq read density, internode explant tissue), and **(D)** genome gap density. X-axes represent the bp dimension of the signals, Y-axes represent scales (*s* in Equation 1). Blue regions indicate positive coefficients and red regions indicate negative coefficients.

**Figure 5:**
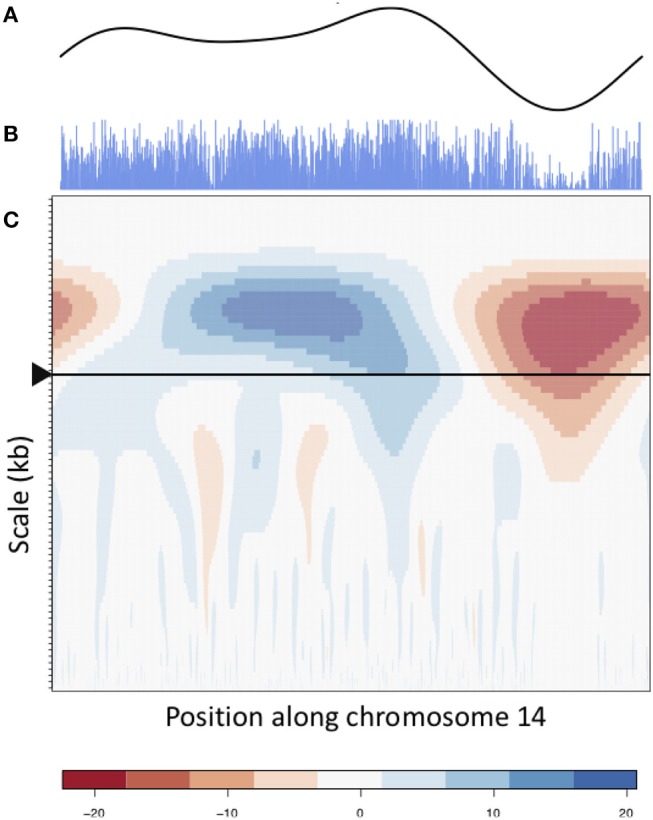
CWT and smooth peaks. CWT landscape of the gene density profile of chromosome 14. **(B)** is the original gene density signal, **(C)** is the CWT coefficient landscape of the signal and **(A)** shows the vector of wavelet coefficients of the scale corresponding to the large scale valley, as shown by the arrow in **(C)**.

The wavelet transform was used to characterize these signals at different scales, identifying peaks of different sizes. Applying the continuous wavelet transform (CWT) to such density signals results in a coefficient landscape for each signal, represented as a heatmap (Spencer et al., 2006) (Figures 4, 5B,C). The x-axis of a coefficient landscape represents the position along the chromosome signal and the y-axis represents the scale, with small scales (high frequency peaks) at the bottom and large scales (low frequency peaks) at the top. A wavelet coefficient is calculated for each signal position and each scale, thus resulting in a landscape. The wavelet coefficient landscapes clearly illustrate the detection of the large scale peaks (blue regions) and large scale valleys (red regions) in the upper half of the landscapes, corresponding to the visible large peaks and valleys of the signals. Plotting the wavelet coefficients at a particular scale shows the smoothed peaks and troughs of the signal at that scale (Figure 5A).

### 3.2. Wavelet Coefficient Landscape Signature of the Centromere

**Figure 6:**
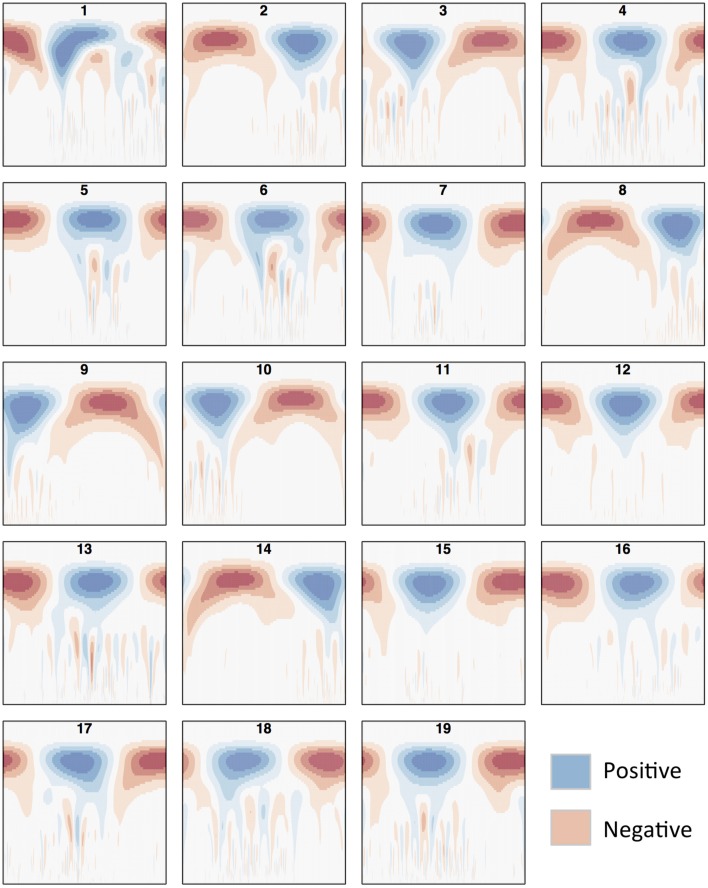
Methylation (internode explant) CWT. Methylation (internode explant tissue) CWT landscapes of each *P. trichocarpa* chromosome. For each heatmap, the x-axis represents position along the chromosome density signal (τ), the y-axis represents scale (*s*) and each entry represents the wavelet coefficient *W*(*s*, τ). Positive coefficients are colored blue and indicate peaks, negative coefficients are colored red and indicate valleys. The “tooth-x-ray” centromeric signature is evident in many chromosomes, consisting of a broad-scale peak encompassing the centromeric/pericentromeric regions, and the lower scale valley within the large peak indicating the centromeric region.

**Figure 7:**
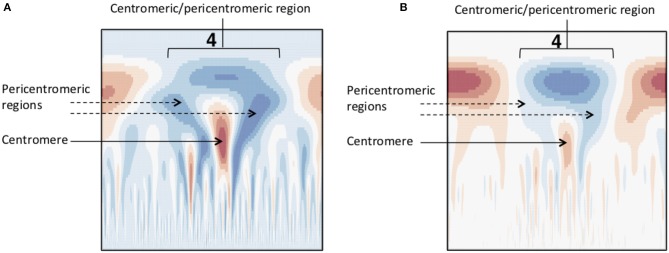
Methylation wavelet signature of centromere. “Tooth-x-ray” centromeric signature for **(A)** SNP density and **(B)** methylation density, consisting of a broad-scale peak encompassing the centromeric/pericentromeric regions, and the lower scale valley within the large peak indicating the centromeric region.

Identification of approximate centromere locations from gene density, SNP density and methylation wavelet landscapes requires knowledge of what patterns to look for. From the literature, we know that studies in *Arabidopsis* have found high methylation in the centromeric/pericentromeric regions (Zhang et al., 2006), and found centromeric regions to be gene-sparse (Copenhaver et al., 1999). Similar conditions were found in *P. trichocarpa* (Vining et al., 2012; Liang et al., 2014). Though centromeric/pericentromeric regions as a whole are highly methylated, it has been found in Maize that the active centromere consists of repeats associated with CENH3 (the modified histone found in the active centromere) and is usually less methylated when compared to the pericentromeric regions (Zhang et al., 2008). A similar pattern can be observed in *Arabidopsis* (Zhang et al., 2006). Figure 6 shows the methylation CWT coefficient landscapes for each chromosome in internode explant tissue. One can clearly see the large-scale peaks in each chromosome indicated by the blue regions near the top of each profile, which correspond to the broad centromeric/pericentromeric regions. In 15 of the 19 chromosomes (chromosomes 1, 2, 4, 5, 6, 7, 8, 9, 10, 13, 14, 16, 17, 18, 19) we see evidence for the lowered methylation in the actual centromere when compared to the pericentromeric regions. In the coefficient landscapes, this is indicated by a medium-scale valley (red area) within and below the center of the large-scale peak, creating a “tooth-X-ray” like pattern (Figure 7). These centromeric wavelet coefficient signatures can also be seen in the methylation profiles of callus, female catkin, male catkin, leaf, phloem, regenerated internode, root and xylem tissues (Figures S26–S34), but are mostly not visible in bud tissue (Figure S25).

SNP density has been found to be higher in the pericentromere in *Arabidopsis* (Ossowski et al., 2010) and lower SNP density has been found in centromere regions in sorghum (Bekele et al., 2013). The SNP wavelet landscapes for all chromosomes all contain the “tooth-X-ray” like shape, indicating a medium-scale valley in SNP density within a large-scale peak (Figure S23). The location of this signature coincides with the large-scale peak in methylation (Figures S26–S34) and valley in gene density (Figure S22), known to be characteristic of centromeric locations. As with the methylation density, this “tooth-X-ray” shape could be indicating the pericentromeric and centromeric regions of the chromosome.

It is important to consider gaps in the assembled genome (Figure S24) when interpreting chromosome density signals, because valleys in a density signal, such as SNP density, could be a meaningful biological signature (such as the centromere), or could be an artifact arising from a gap in the genome. Observing the density signals for all chromosomes (Figures S2–S20) and their wavelet landscapes (Figures S22–S34) one can see that in a few chromosomes, (for example, chromosome 18) the largest genome gap co-locates with the largest valley in SNP density. However, this is not true for all chromosomes. The locations of highest genome gap density do not always coincide with the largest valley in SNP density, for example, in chromosome 12 (Figure S21), and the largest genome gaps do not always correspond to approximate centromere locations. Thus, the tooth-X-ray shape cannot be purely driven by genome gaps, and, as such, does not appear to be an artifact.

### 3.3. Prediction of Centromere Position From Wavelet Coefficients

**Figure 8:**
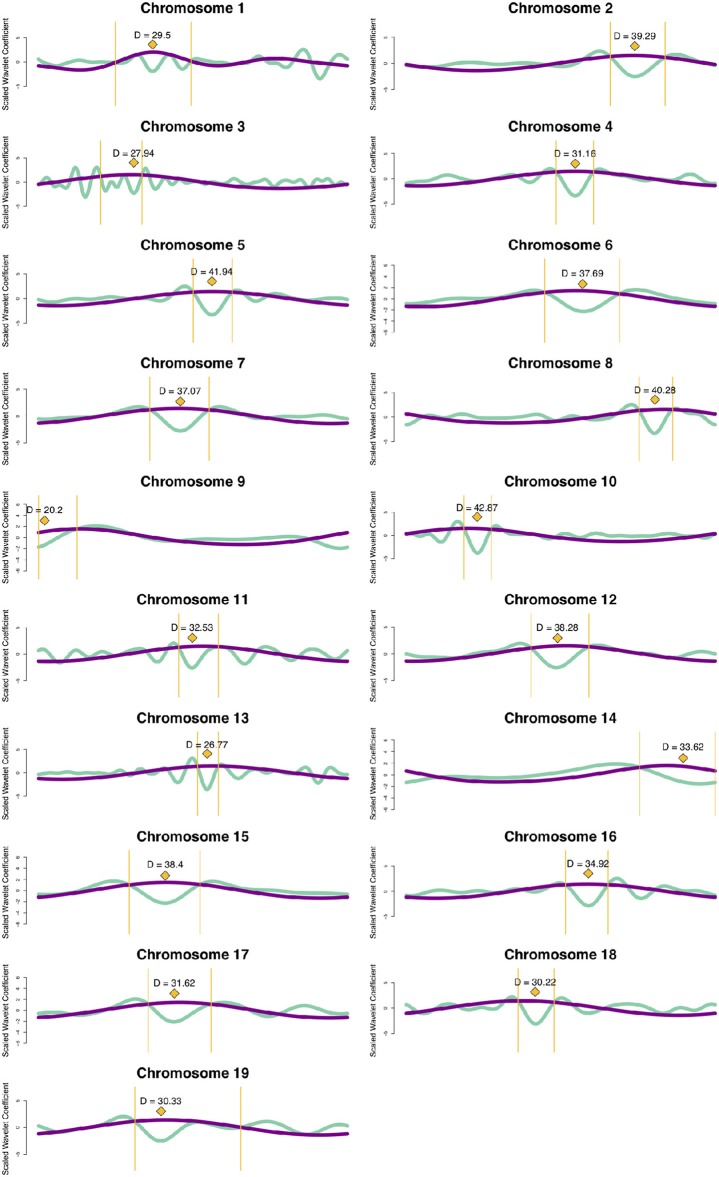
Centromere positions. Line plots for each chromosome of methylation wavelet coefficients (internode explant tissue) at pericentromeric scale (purple lines) and SNP density wavelet coefficients at centromeric scale (green lines). Yellow diamonds represent the putative centromeric location, calculated as the point of maximum difference between the wavelet coefficients at these two scales. Yellow bars indicate the general centromeric region as the points of intersection between the two curves on either side of the centromeric region. The maximum difference (*D*) between the unscaled wavelet coefficients used to determine the putative centromeric location (yellow diamonds) are shown for each chromosome. See Methods for further details.

Based on the knowledge of centromere signatures in the literature, and the CWT landscapes of gene, SNP and methylation profiles, we attempted to locate the position of the centromere on each *P. trichocarpa* chromosome by computationally identifying the characteristic tooth-X-ray shape in the CWT landscapes. Briefly, for each chromosome, we calculate the CWT of the scaled SNP density and methylation profiles, resulting in two coefficient landscapes. We identify the pericentromeric scale as the scale at which we find the maximum wavelet coefficient in the upper third of the methylation landscape, and identify the borders of the pericentromeric region as the zeroes of the wavelet vector on either side of the maximum coefficient. We then identify the centromeric scale as the minimum wavelet coefficient in the SNP wavelet landscape within the borders of the pericentromeric region, and then consider the approximate center of the centromere location to be the point of maximum difference between the methylation wavelet coefficients at the pericentromere scale and SNP wavelet coefficients at the centromere scale (Figure 8, Figure S1; Supplementary Note S1), and the general centromeric region borders as the points of intersection between the these two vectors on either side of the center (Table S1, yellow bars in Figure 8).

**Figure 9:**
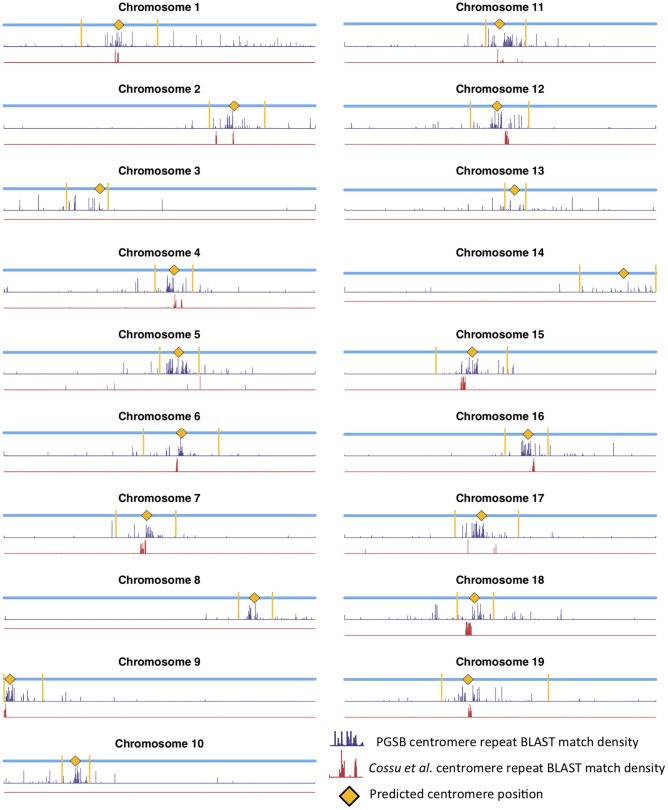
Centromere positions and centromere repeats. Putative centromere positions (yellow diamonds) identified as in Figure 8 using methylation and SNP wavelet coefficients, as well as the density of BLAST matches of plant centromere repeat sequences (navy bars) and putative *P. trichocarpa* centromere repeat sequences (Cossu et al., 2012) (red bars).

Mapping centromere repeats from various plants from the PGSB Repeat Element Catalog (Nussbaumer et al., 2012) as well as repeat sequences which were found to identify centromeres on certain *P. trichocarpa* chromosomes in a previous assembly (Cossu et al., 2012) using BLAST were consistent with the locations of centromeres identified using wavelet coefficients. Predicted centromere positions aligned well with the density profiles of repeat sequence BLAST hits, indicating that our centromere prediction strategy is likely identifying valid centromere positions (Figure 9). The wavelet-based centromere identification through the use of multiple lines of evidence allows us to be more certain of centromeric regions, and also allows more specific locations to be identified than can be done by simply looking at repeat density, which map to broad regions of the genome. Layering multiple data types allows for the identification of putative centromere positions based on multiple lines of evidence, and thus, allows one to be more certain of their location.

**Figure 10:**
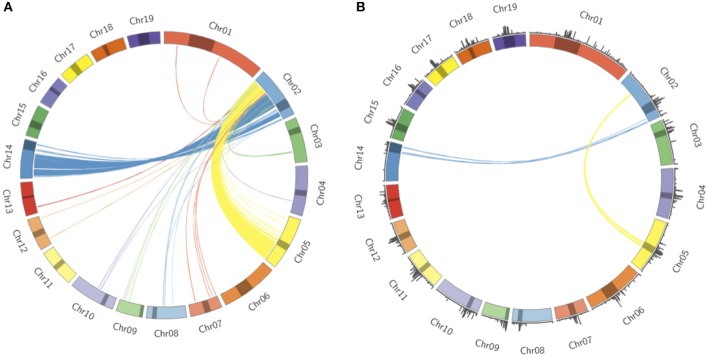
Syntenic blocks and ancestral centromeric DNA. **(A)** Circos plot representing syntenic blocks involving homologous segments of DNA between chromosome two and other *P. trichocarpa* chromosomes. Chromosome lengths are represented on the ideogram with the predicted centromeric/pericentromeric regions shown as dark highlights. Links between chromosomes indicate homologous chromosome regions, and are colored according to the source chromosome, with chromosome 2 being the target chromosome. **(B)** Syntenic blocks which overlap with predicted centromeric/pericentromeric regions on source chromosomes. PGSB centromere repeat densities from Figure 9 are included as a bar chart on the ideogram.

*Populus trichocarpa* chromosomes contain homologous genome blocks, presumed to be derived from the salicoid genome duplication (Tuskan et al., 2006). Looking at the positions of predicted centromeres in Figures 8, 9, some paralogous chromosomes (see Tuskan et al., 2006) appear to have similar centromeric positions (for example, chromosomes 8 and 10, and chromosomes 12 and 15). This suggests that the current centromere positions potentially predate the salicoid duplication event. One can see in Figure 9 that certain PGSB peaks exist outside of predicted centromeric regions, suggesting centromere-like repeat sequences outside of the predicted active centromeric regions. These peaks outside of centromeric regions tend to overlap with syntenic blocks arising from a genome rearrangement involving a centromeric region (Figure 10, Figures S35, S36). For example, Figure 10A shows circos plots of all the syntenic blocks/homologous chromosome regions centered around chromosome 2. Centromeric regions predicted are shown as highlights on the chromosome ideogram. Visualizing only the syntenic blocks which overlap with centromeric regions (Figure 10B) provides information on the fate of active centromeres/centromeric DNA post-rearrangement. One can also see evidence for cases where the active centromere of a given chromosome segment was maintained after the chromosome rearrangement. The PGSB density plots from Figure 9 are shown as bar plots along the chromosome ideogram. Similar plots for other chromosomes can be seen in Figures S35, S36. These PGSB peaks representing centromere-like sequences outside of active centromere locations align well with syntenic blocks arising from centromeric locations, and can thus be interpreted as pieces of historic centromeric DNA from a genome duplication and subsequent genome rearrangement, known to occur in the history of *P. trichocarpa*.

### 3.4. Co-evolution of Putative CENH3 With Centromeric Sequences

The histone CENH3 epigenetically defines centromere position, and replaces normal histone H3 in the nucleosomes at the centromere (Watts et al., 2016). Silencing of this gene in *Arabidopsis* has been found to cause dwarfism, reduced mitotic divisions and sterility (Lermontova et al., 2011). CENH3 has been found to be adaptively evolving in *Arabidopsis* (Talbert et al., 2002). Analysis of CENH3 in various *Brassicaceae* showed that it is evolving adaptively at various sites which are potentially in contact with the centromeric DNA (Cooper and Henikoff, 2004). There is thus the hypothesis that CENH3 is co-evolving with the sequence of the centromere (Henikoff et al., 2001; Cooper and Henikoff, 2004). In a study involving a *A. thaliana* CENH3-null mutant expressing a *Zea mays* CENH3, it was found that while the *Zea mays* CENH3 localized to the same locations as endogenous *A. thaliana* CENH3, the *Z. mays* CENH3 centromeres were weaker, and resulted in genome elimination in crosses with wild-type *A. thaliana* (Maheshwari et al., 2017). Thus, the sequence of CENH3 could potentially have an impact on the strength of the centromere.

**Figure 11:**
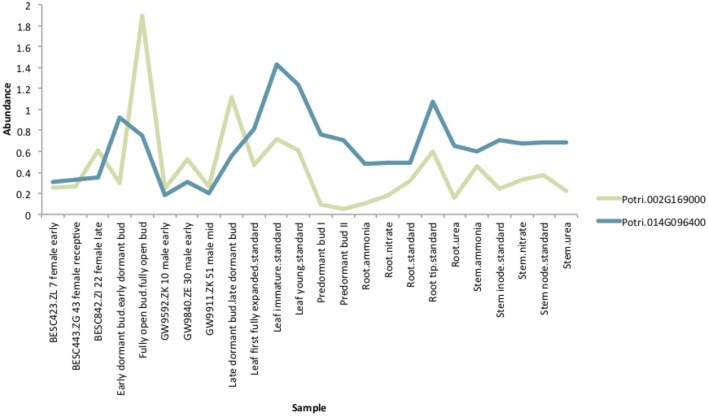
CENH3 expression. Expression levels of putative *P. trichocarpa* CENH3 genes, Potri.002G169000 and Potri.014G096400. Expression data obtained from PhytoMine on Phytozome (Goodstein et al., 2012).

If the hypothesis of co-evolution between the CENH3 and centromeric sequences is true, one would expect to see correlations between Single Nucleotide Polymorphisms (SNPs) in *P. trichocarpa* CENH3 and *P. trichocarpa* centromeric regions. CENH3 is mostly a single copy in diploids, such as *Arabidopsis* (Watts et al., 2016) but there are some species that contain more than one copy. Wheat has two distinct copies of CENH3, and they seem to be evolutionarily divergent. They have different expression patterns, and one of them shows positive selection (Yuan et al., 2015). We identified two putative CENH3 genes in *P. trichocarpa* (Potri.014G096400 on chromosome 14 and Potri.002G169000 on chromosome 2) as BLAST (Altschul et al., 1990) matches of *A. thaliana* CENH3 (AT1G01370, for BLAST results see Table S2). It is interesting to note that chromosomes 2 and 14 are salicoid duplication paralogs. Of these two genes, Potri.014G096400 was annotated as being similar to a CENH3 gene, whereas Potri.002G169000 had no functional annotations. RNA-seq and EST information on Phytozome (Goodstein et al., 2012) confirmed that both of these genes are expressed (Figure S37). Expression information of these genes in the *P. trichocarpa* gene atlas on PhytoMine (Goodstein et al., 2012; Kalderimis et al., 2014) showed that the expression of these two genes varies across tissues, however, they are not co-expressed with one another (Figure 11). Both Potri.014G096400 and Potri.002G169000 genome sequences had multiple hits with CENH3 genes when BLASTed on NCBI.

**Figure 12:**
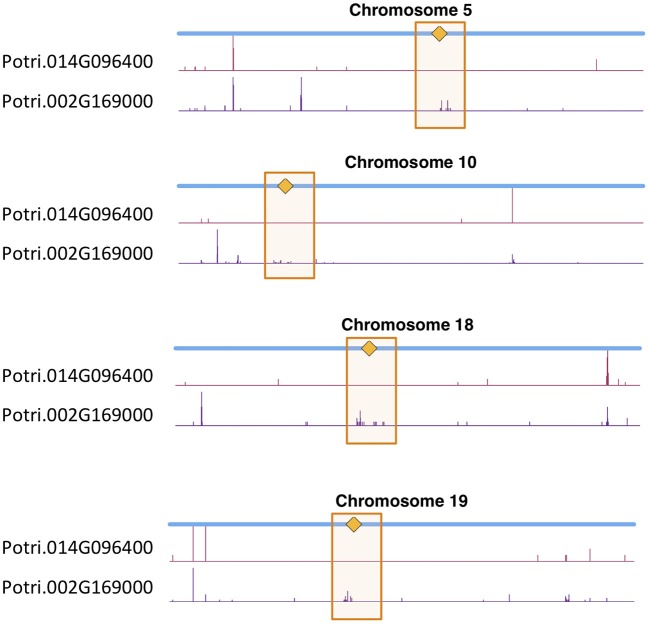
CENH3 co-evolution. SNP density profiles across a selection of chromosomes involving SNPs which correlate with SNPs in putative CENH3 genes, Potri.002G169000 and Potri.014G096400 across a population of *P. trichocarpa* genotypes. One can clearly see the clusters of SNPs in the centromeric regions which are correlating with SNPs within these CENH3 genes.

We determined correlations between all pairs of ~10,000,000 high confidence SNPs in a population of 882 *P. trichocarpa* genotypes using the CCC metric (Climer et al., 2014a,b; Joubert et al., 2017) and extracted SNPs within Potri.014G096400 and Potri.002G169000 that had correlations with SNPs elsewhere in the genome (Tables S3, S4). When using a call rate constraint minimum of a 100 called alleles (~5%), a minimum overlap of 100 non-missing alleles in SNP correlations and requiring a minor allele frequency (MAF) ≥0.01, we find concentrations of SNPs in the centromeric region of various chromosomes which are correlated with SNPs in Potri.002G169000 (Figure 12, Figure S38). We thus find strong evidence for the co-evolution for CENH3 with the centromeric sequences.

**Figure 13:**
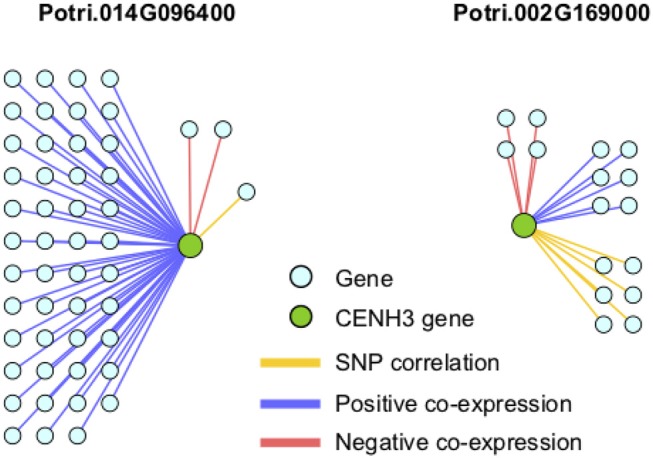
CENH3 gene correlations. Correlations of *P. trichocarpa* CENH3 genes (green circles) with other genes (aqua circles), including positive co-expression (blue), negative co-expression (red), and SNP correlations (yellow).

In particular, it appears that Potri.002G169000 seems to have a co-evolution signature with the centromere, much more so than Potri.014G096400, in that Potri.002G169000 contained SNPs correlating with 13 out of 19 centromeric regions, whereas Potri.014G096400 contained SNPs correlating with 5 out of 19 centromeric regions (centromeric regions in Table S1). While both Potri.002G169000 and Potri.014G096400 on average have more mutations than other *P. trichocarpa* histones (an expected phenomenon as CENH3 histones accumulate mutations faster than normal histones, as mentioned in Maheshwari et al., 2015), Potri.002G169000 contains more mutations than Potri.014G096400 (Figure S39; Table S5). Potri.014G096400 is also co-expressed with various other non-CENH3 histones, as well as a histone deacetylase and a histone methyltransferase on PhytoMine (Goodstein et al., 2012; Kalderimis et al., 2014) (Table S3), and the correlation neighborhood of Potri.002G169000 and Potri.014G096400 do not overlap at all (Figure 13; Tables S6, S7).

**Figure 14:**
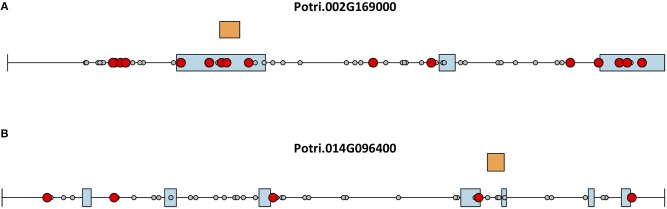
CENH3 mutations. SNPs in putative *P. trichocarpa* CENH3 genes **(A)** Potri.002G169000 and **(B)** Potri.014G096400. Exons (blue boxes) for Potri.014G096400 were determined from the v3.0 genome annotation on Phytozome (Goodstein et al., 2012), and from mapped ESTs on Phytozome JBrowse (Skinner et al., 2009; Goodstein et al., 2012) for Potri.002G169000. Gray circles represent SNPs, red circles represent SNPs that correlate with SNPs in centromeric regions. Orange rectangles indicate the location of the histone domain as determined using NCBI CDScan.

This seems to suggest that these two genes are functionally divergent. Given the facts that Potri.002G169000 has strong co-evolution signatures with the centromere (Figure 12) and Potri.014G096400 is co-expressing with non-CENH3 histones, one could hypothesize that Potri.002G169000 (a previously unannotated gene) is the primary functioning CENH3 in *P. trichocarpa* while Potri.014G096400 could be functioning more like a normal histone. If one looks at the position of SNPs within Potri.002G169000 and Potri.014G096400, it is evident that Potri.002G169000 contains more SNPs in transcribed regions of the gene that correlate with centromeric regions (Figure 14). In addition, Potri.002G169000 contains more SNPs in/near the histone domain that correlate with the centromere, when compared to Potri.014G096400. Potri.002G169000 also has more of the expected structure for a CENH3 gene, containing the histone domain in the C terminal, and having a variable N terminal domain (Watts et al., 2016).

Based on these various lines of evidence, we suggest that the previously unannotated Potri.002G169000 is the primary functioning CENH3 gene in *P. trichocarpa*.

### 3.5. Concluding Remarks

In this study we performed wavelet-based signal processing of multiple, heterogeneous data types to identify centromere positions and properties in *P. trichocarpa*. We found centromeres to be in gene-sparse regions, and found centromeric/pericentromeric regions to hypermethylated relative to the rest of the chromosomes, and found centromeric DNA to be hypomethylated relative to pericentromeric regions in many chromosomes across various tissues. The “tooth-X-ray” wavelet signature was identified as a characteristic signature of the centromere in the wavelet landscapes of SNP density profiles.

The use of wavelet coefficients allowed us to identify the approximate centromeric locations. These locations were supported by mapping of repeat sequences, and could be further validated through experimental techniques, such as ChIP (chromatin immunoprecipitation)-Seq. We also found evidence for the co-evolution of the sequence of the centromere-specific histone CENH3 with the sequences of the centromere on many chromosomes. In particular, we found that the previously unannotated gene Potri.002G169000 is the most likely candidate for an active, centromere-co-evolving CENH3 gene in *P. trichocarpa* and not the currently annotated CENH3 gene, Potri.014G096400.

This study illustrates how through integrating multiple sources of data, one can arrive at a more comprehensive understanding of the system one is investigating. In this case, we have produced at a more reliable and detailed characterization of centromere location in *P. trichocarpa*, involving information from multiple ‘omics data layers and providing information about centromeric signatures derived from multiple sources. In order to extend these analyses to produce an automated centromere prediction approach applicable to other species, further testing and validation will be required. The wavelet-based approach would need to be applied to multiple species, and validated against exact centromeric locations determined using ChIP-seq.

This study illustrated the utility of wavelet-based signal processing of genomic signals to identify structural characteristics of chromosomes. While this study made primary use of the larger-scale wavelet coefficients, we would recommend the use of the smaller scale wavelet coefficients to investigate smaller-scale structural characteristics, such as nucleosome occupancy.

## Author Contributions

DW and DJ conceived of and designed the study. DW performed the analysis, developed the wavelet centromere identification strategy, interpreted the results, and wrote the manuscript. GT lead the sequencing of *Populus* genotypes. SD and DM-S performed the SNP calling. JS and AS contributed the genome sequence and transcriptome expression analysis. MS mapped gene expression atlas reads and calculated gene expression TPM values. WJ developed the parallel GPU CCC metric code. DW, DJ, GT, DM-S, and AS edited the manuscript.

### Conflict of Interest Statement

The authors declare that the research was conducted in the absence of any commercial or financial relationships that could be construed as a potential conflict of interest.

## References

[B1] Altschul S. F. Gish W. Miller W. Myers E. W. Lipman D. J. (1990). Basic local alignment search tool. J. Mol. Biol. 215, 403–410. 2231712 10.1016/S0022-2836(05)80360-2

[B2] Barnett D. W. Garrison E. K. Quinlan A. R. Strömberg M. P. Marth G. T. (2011). BamTools: a C++ API and toolkit for analyzing and managing BAM files. Bioinformatics 27, 1691–1692. 10.1093/bioinformatics/btr174 21493652 PMC3106182

[B3] Bekele W. A. Wieckhorst S. Friedt W. Snowdon R. J. (2013). High-throughput genomics in sorghum: from whole-genome resequencing to a snp screening array. Plant Biotechnol. J. 11, 1112–1125. 10.1111/pbi.12106 23919585

[B4] Climer S. Templeton A. R. Zhang W. (2014a). Allele-specific network reveals combinatorial interaction that transcends small effects in Psoriasis GWAS. PLoS Comput. Biol. 10:e1003766. 10.1371/journal.pcbi.1003766 25233071 PMC4168982

[B5] Climer S. Yang W. Fuentes L. Dávila-Román V. G. Gu C. C. (2014b). A custom correlation coefficient (CCC) approach for fast identification of multi-SNP association patterns in genome-wide SNPs data. Genet. Epidemiol. 38, 610–621. 10.1002/gepi.21833 25168954 PMC4190009

[B6] Constantine W. Hesterberg T. Wittkowski K. Song T. Kaluzny S. (2016). splus2R: Supplemental S-PLUS Functionality in R. R package version 1.2-2.

[B7] Constantine W. Percival D. (2016). wmtsa: Wavelet Methods for Time Series Analysis. R package version 2.0-2.

[B8] Cooper J. L. Henikoff S. (2004). Adaptive evolution of the histone fold domain in centromeric histones. Mol. Biol. Evol. 21, 1712–1718. 10.1093/molbev/msh179 15175412

[B9] Copenhaver G. P. Nickel K. Kuromori T. Benito M.-I. Kaul S. Lin X.. (1999). Genetic definition and sequence analysis of arabidopsis centromeres. Science 286, 2468–2474. 10617454 10.1126/science.286.5449.2468

[B10] Cossu R. M. Buti M. Giordani T. Natali L. Cavallini A. (2012). A computational study of the dynamics of LTR retrotransposons in the populus trichocarpa genome. Tree Genet. Genomes 8, 61–75. 10.1007/s11295-011-0421-3

[B11] Danecek P. Auton A. Abecasis G. Albers C. A. Banks E. DePristo M. A.. (2011). The variant call format and VCFtools. Bioinformatics 27, 2156–2158. 10.1093/bioinformatics/btr330 21653522 PMC3137218

[B12] Evans L. M. Slavov G. T. Rodgers-Melnick E. Martin J. Ranjan P. Muchero W.. (2014). Population genomics of *Populus trichocarpa* identifies signatures of selection and adaptive trait associations. Nat. Genet. 46, 1089–1096. 10.1038/ng.3075 25151358

[B13] Feng C. Liu Y. Su H. Wang H. Birchler J. Han F. (2015). Recent advances in plant centromere biology. Sci. China Life Sci. 58, 240–245. 10.1007/s11427-015-4818-3 25682396

[B14] Furuyama S. Biggins S. (2007). Centromere identity is specified by a single centromeric nucleosome in budding yeast. Proc. Natl. Acad. Sci. U.S.A. 104, 14706–14711. 10.1073/pnas.0706985104 17804787 PMC1976213

[B15] Gomez-Cabrero D. Abugessaisa I. Maier D. Teschendorff A. Merkenschlager M. Gisel A. J., (2014). Data integration in the era of omics: current and future challenges. BMC Syst. Biol. 8:I1. 10.1186/1752-0509-8-S2-I1 25032990 PMC4101704

[B16] Goodstein D. M. Shu S. Howson R. Neupane R. Hayes R. D. Fazo J.. (2012). Phytozome: a comparative platform for green plant genomics. Nucleic Acids Res. 40, D1178–D1186. 10.1093/nar/gkr944 22110026 PMC3245001

[B17] Grigoriev I. V. Nordberg H. Shabalov I. Aerts A. Cantor M. Goodstein D.. (2011). The genome portal of the Department of Energy Joint Genome Institute. Nucleic Acids Res. 40, 1–7. 10.1093/nar/gkr947 22110030 PMC3245080

[B18] Haug-Baltzell A. Stephens S. A. Davey S. Scheidegger C. E. Lyons E. (2017). SynMap2 and SynMap3D: web-based whole-genome synteny browsers. Bioinformatics 33, 2197–2198. 10.1093/bioinformatics/btx144 28334338

[B19] Henikoff S. Ahmad K. Malik H. S. (2001). The centromere paradox: stable inheritance with rapidly evolving dNA. Science 293, 1098–1102. 10.1126/science.1062939 11498581

[B20] Joubert W. Nance J. Climer S. Weighill D. Jacobson D. (2017). Parallel accelerated custom correlation coefficient calculations for genomics applications. Parallel Comput. 84, 15–23. 10.1016/j.parco.2019.02.003

[B21] Kalderimis A. Lyne R. Butano D. Contrino S. Lyne M. Heimbach J.. (2014). Intermine: extensive web services for modern biology. Nucleic Acids Res. 42, W468–W472. 10.1093/nar/gku301 24753429 PMC4086141

[B22] Krzywinski M. I. Schein J. E. Birol I. Connors J. Gascoyne R. Horsman D.. (2009). Circos: an information aesthetic for comparative genomics. Genome Res. 19, 1639–1645. 10.1101/gr.092759.109 19541911 PMC2752132

[B23] Leavey C. James M. Summerscales J. Sutton R. (2003). An introduction to wavelet transforms: a tutorial approach. Insight Non Destruct. Testing Condit. Monit. 45, 344–353. 10.1784/insi.45.5.344.52875

[B24] Lermontova I. Koroleva O. Rutten T. Fuchs J. Schubert V. Moraes I.. (2011). Knockdown of CENH3 in arabidopsis reduces mitotic divisions and causes sterility by disturbed meiotic chromosome segregation. Plant J. 68, 40–50. 10.1111/j.1365-313X.2011.04664.x 21635586

[B25] Li H. Handsaker B. Wysoker A. Fennell T. Ruan J. Homer N.. (2009). The sequence alignment/map format and SAMtools. Bioinformatics 25, 2078–2079. 10.1093/bioinformatics/btp352 19505943 PMC2723002

[B26] Liang D. Zhang Z. Wu H. Huang C. Shuai P. Ye C.-Y.. (2014). Single-base-resolution methylomes of populus trichocarpa reveal the association between dna methylation and drought stress. BMC Genet. 15:S9. 10.1186/1471-2156-15-S1-S9 25080211 PMC4118614

[B27] Lyons E. Pedersen B. Kane J. Freeling M. (2008). The value of nonmodel genomes and an example using SynMap within CoGe to dissect the hexaploidy that predates the rosids. Trop. Plant Biol. 1, 181–190. 10.1007/s12042-008-9017-y

[B28] Machado J. T. Costa A. C. Quelhas M. D. (2011). Wavelet analysis of human DNA. Genomics 98, 155–163. 10.1016/j.ygeno.2011.05.010 21672622

[B29] Maheshwari S. Ishii T. Brown C. T. Houben A. Comai L. (2017). Centromere location in arabidopsis is unaltered by extreme divergence in CENH3 protein sequence. Genome Res. 27, 471–478. 10.1101/gr.214619.116 28223399 PMC5340974

[B30] Maheshwari S. Tan E. H. West A. Franklin F. C. H. Comai L. Chan S. W. (2015). Naturally occurring differences in CENH3 affect chromosome segregation in zygotic mitosis of hybrids. PLoS Genet. 11:e1004970. 10.1371/journal.pgen.1004970 25622028 PMC4314295

[B31] Marchler-Bauer A. Bryant S. H. (2004). CD-search: protein domain annotations on the fly. Nucleic Acids Res. 32(Suppl._2), W327–W331. 10.1093/nar/gkh454 15215404 PMC441592

[B32] Marchler-Bauer A. Derbyshire M. K. Gonzales N. R. Lu S. Chitsaz F. Geer L. Y.. (2014). CDD: Ncbi's conserved domain database. Nucleic Acids Res. 43, D222–D226. 10.1093/nar/gku1221 25414356 PMC4383992

[B33] Marchler-Bauer A. Lu S. Anderson J. B. Chitsaz F. Derbyshire M. K. DeWeese-Scott C.. (2010). CDD: a conserved domain database for the functional annotation of proteins. Nucleic Acids Res. 39(Suppl._1), D225–D229. 10.1093/nar/gkq1189 21109532 PMC3013737

[B34] McCormick R. F. Truong S. K. Sreedasyam A. Jenkins J. Shu S. Sims D.. (2017). The Sorghum bicolor reference genome: improved assembly and annotations, a transcriptome atlas, and signatures of genome organization. bioRxiv 110593. 10.1101/110593 29161754

[B35] Mehrotra S. Goyal V. (2014). Repetitive sequences in plant nuclear DNA: types, distribution, evolution and function. Genomics Proteomics Bioinformatics 12, 164–171. 10.1016/j.gpb.2014.07.003 25132181 PMC4411372

[B36] Neph S. Kuehn M. S. Reynolds A. P. Haugen E. Thurman R. E. Johnson A. K.. (2012). BEDOPS: high-performance genomic feature operations. Bioinformatics 28, 1919–1920. 10.1093/bioinformatics/bts277 22576172 PMC3389768

[B37] Neuwirth E. (2014). RColorBrewer: ColorBrewer Palettes. R package version 1.1-2.

[B38] Nordberg H. Cantor M. Dusheyko S. Hua S. Poliakov A. Shabalov I.. (2014). The genome portal of the Department of Energy Joint Genome Institute: 2014 updates. Nucleic Acids Res. 42, D26–D31. 10.1093/nar/gkt1069 24225321 PMC3965075

[B39] Nussbaumer T. Martis M. M. Roessner S. K. Pfeifer M. Bader K. C. Sharma S.. (2012). MIPS plantsDB: a database framework for comparative plant genome research. Nucleic Acids Res. 41, D1144–D1151. 10.1093/nar/gks1153 23203886 PMC3531202

[B40] Nychka D. Furrer R. Paige J. Sain S. (2017). Fields: Tools for Spatial Data. R package version 9.6.

[B41] O'Connor C. (2008). Chromosome segregation in mitosis: the role of centromeres. Nat. Educ. 1:28.

[B42] Ossowski S. Schneeberger K. Lucas-Lledó J. I. Warthmann N. Clark R. M. Shaw R. G.. (2010). The rate and molecular spectrum of spontaneous mutations in arabidopsis thaliana. Science 327, 92–94. 10.1126/science.1180677 20044577 PMC3878865

[B43] Percival D. B. Walden A. T. (2006). Wavelet Methods for Time Series Analysis, Vol. 4. New York, NY: Cambridge University Press.

[B44] Pinosio S. Giacomello S. Faivre-Rampant P. Taylor G. Jorge V. Le Paslier M. C.. (2016). Characterization of the poplar pan-genome by genome-wide identification of structural variation. Mol. Biol. Evol. 33, 2706–2719. 10.1093/molbev/msw161 27499133 PMC5026262

[B45] Purcell S. Neale B. Todd-Brown K. Thomas L. Ferreira M. A. Bender D.. (2007). Plink: a tool set for whole-genome association and population-based linkage analyses. Am. J. Hum. Genet. 81, 559–575. 10.1086/519795 17701901 PMC1950838

[B46] Quinlan A. R. (2014). Bedtools: the swiss-army tool for genome feature analysis. Curr. Protoc. Bioinformatics 47, 11–12. 10.1002/0471250953.bi1112s47 25199790 PMC4213956

[B47] R Core Team (2015). R: A Language and Environment for Statistical Computing. Vienna: R Foundation for Statistical Computing.30628467

[B48] RStudio Team (2016). RStudio: Integrated Development Environment for R. Boston, MA: RStudio, Inc.

[B49] Shannon P. Markiel A. Ozier O. Baliga N. S. Wang J. T. Ramage D.. (2003). Cytoscape: a software environment for integrated models of biomolecular interaction networks. Genome Res. 13, 2498–2504. 10.1101/gr.1239303 14597658 PMC403769

[B50] Skinner M. E. Uzilov A. V. Stein L. D. Mungall C. J. Holmes I. H. (2009). JBrowse: A next-generation genome browser. Genome Res. 19, 1630–1638. 10.1101/gr.094607.109 19570905 PMC2752129

[B51] Slavov G. T. DiFazio S. P. Martin J. Schackwitz W. Muchero W. Rodgers-Melnick E.. (2012). Genome resequencing reveals multiscale geographic structure and extensive linkage disequilibrium in the forest tree *Populus trichocarpa*. New Phytol. 196, 713–725. 10.1111/j.1469-8137.2012.04258.x 22861491

[B52] Spencer C. C. Deloukas P. Hunt S. Mullikin J. Myers S. Silverman B.. (2006). The influence of recombination on human genetic diversity. PLoS Genet. 2:e148. 10.1371/journal.pgen.0020148 17044736 PMC1575889

[B53] Talbert P. B. Masuelli R. Tyagi A. P. Comai L. Henikoff S. (2002). Centromeric localization and adaptive evolution of an arabidopsis histone H3 variant. Plant Cell 14, 1053–1066. 10.1105/tpc.010425 12034896 PMC150606

[B54] Tange O. (2011). GNU parallel-the command-line power tool. USENIX Mag. 36, 42–47.

[B55] Tuskan G. Slavov G. DiFazio S. Muchero W. Pryia R. Schackwitz W. (2011). *Populus* resequencing: towards genome-wide association studies. BMC Proc. 5:I21 10.1186/1753-6561-5-S7-I21

[B56] Tuskan G. A. Difazio S. Jansson S. Bohlmann J. Grigoriev I. Hellsten U.. (2006). The Genome of Black Cottonwood, *Populus trichocarpa* (Torr. & Gray). Science 313, 1596–1604. 10.1126/science.1128691 16973872

[B57] Vining K. J. Pomraning K. R. Wilhelm L. J. Priest H. D. Pellegrini M. Mockler T. C.. (2012). Dynamic DNA cytosine methylation in the *Populus trichocarpa* genome: tissue-level variation and relationship to gene expression. BMC Genomics 13:27. 10.1186/1471-2164-13-27 22251412 PMC3298464

[B58] Watts A. Kumar V. Bhat S. R. (2016). Centromeric histone H3 protein: from basic study to plant breeding applications. J. Plant Biochem. Biotechnol. 25, 339–348. 10.1007/s13562-016-0368-4

[B59] Weighill D. A. Jones P. Shah M. Ranjan P. Muchero W. Schmutz J. (2018). Pleiotropic and epistatic network-based discovery: Integrated networks for target gene discovery. Front. Energy Res. 6:30 10.3389/fenrg.2018.00030

[B60] Wu Q. Castleman K. R. (2000). “Automated chromosome classification using wavelet-based band pattern descriptors,” in Computer-Based Medical Systems, 2000. CBMS 2000. Proceedings. 13th IEEE Symposium on (Houston, TX: IEEE), 189–194.

[B61] Yuan J. Guo X. Hu J. Lv Z. Han F. (2015). Characterization of two CENH3 genes and their roles in wheat evolution. New Phytol. 206, 839–851. 10.1111/nph.13235 25557089

[B62] Zhang W. Lee H.-R. Koo D.-H. Jiang J. (2008). Epigenetic modification of centromeric chromatin: hypomethylation of dna sequences in the cenh3-associated chromatin in arabidopsis thaliana and maize. Plant Cell 20, 25–34. 10.1105/tpc.107.057083 18239133 PMC2254920

[B63] Zhang X. Yazaki J. Sundaresan A. Cokus S. Chan S. W.-L. Chen H.. (2006). Genome-wide high-resolution mapping and functional analysis of dna methylation in arabidopsis. Cell 126, 1189–1201. 10.1016/j.cell.2006.08.003 16949657

