# Supplementary material for: Wavelet-Based Genomic Signal Processing for Centromere Identification and Hypothesis Generation
Source: Front Genet. 2019 May 31;10:487. doi: 10.3389/fgene.2019.00487 (PMC6554479; doi:10.3389/fgene.2019.00487)
Supplement: Supplementary file 1 [file Data_Sheet_1.PDF]

Supplementary Material:
Wavelet-based Genomic Signal Processing for Centromere Identification and Hypothesis Generation

SUPPLEMENTARY NOTES

Note S1: Wavelet-based Centromere Identification

For the demonstrated centromere identification, we used the methylation density signal from internode explant tissue. Centromere identification was performed independently for each chromosome. SNP density and methylation density signals were mean centered (mean = 0) scaled to have standard deviation = 1. The continuous wavelet transform (CWT) was then performed on the scaled signal vectors. The methylation wavelet landscape was then used to identify the "general" centromeric/pericentromeric region of the chromosome. This gives us a general region of the chromosome to start looking for the "tooth-X-ray" shape, since identifying the centromere "valley" in the SNP wavelet landscapes by looking for minimum wavelet coefficients could instead identify valleys in other parts of the chromosome (see for example in chromosome 1, Figure S23, the minimum wavelet coefficient does not appear in the middle of the "tooth-X-ray" shape.)

The procedure for identifying centromere positions is as follows:

1. Identify the position of the maximum wavelet coefficient in the upper third of the methylation landscape. We call the scale at which this maximum occurs the "pericentromere scale".
2. Find the putative pericentromere borders as the zeros on their side of this maximum. If the centromere is near the end of the chromosome, the one "pericentromere border" might be the edge of the chromosome.
3. Identify the the minimum coefficient in the lower two thirds of the SNP wavelet landscape, between the approximate pericentromere borders. We call the scale at which this minimum occurs the "centromere scale".
4. Extract the SNP wavelet coefficient vector at centromere scale and the methylation wavelet coefficient vector at pericentromere scale.
5. Mean center (mean = 0) and scale these vectors to have standard deviation 1, and find the approximate centromere location as the position of maximum difference between these two vectors.

This manuscript has been authored by UT-Battelle, LLC under Contract No. DE-AC05-00OR22725 with the U.S. Department of Energy. The United States Government retains and the publisher, by accepting the article for publication, acknowledges that the United States Government retains a non-exclusive, paid-up, irrevocable, worldwide license to publish or reproduce the published form of this manuscript, or allow others to do so, for United States Government purposes. The Department of Energy will provide public access to these results of federally sponsored research in accordance with the DOE Public Access Plan (http://energy.gov/downloads/doe-public-access-plan).

SUPPLEMENTARY FIGURES

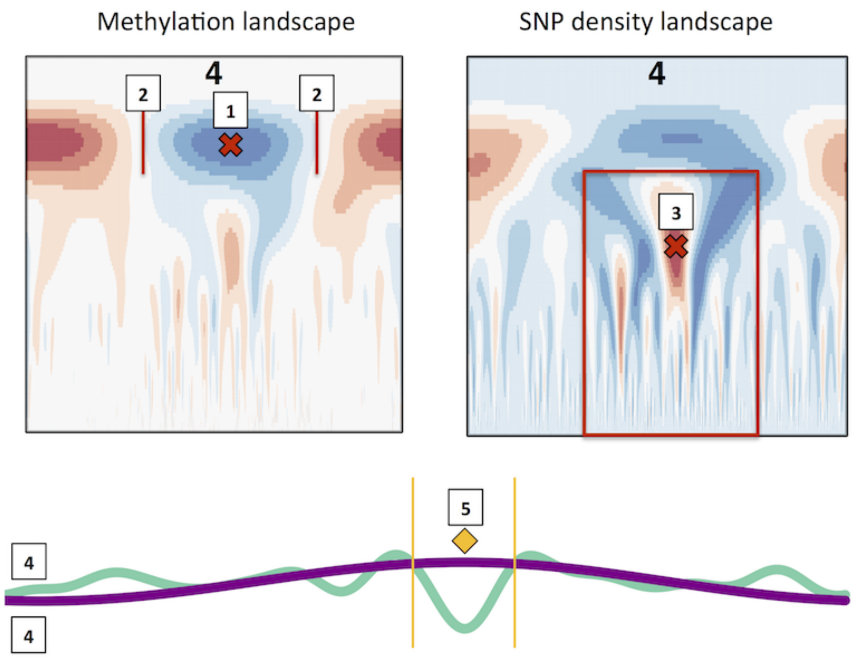

Figure S1: Approach for identifying centromere positions from wavelet coefficient landscapes.

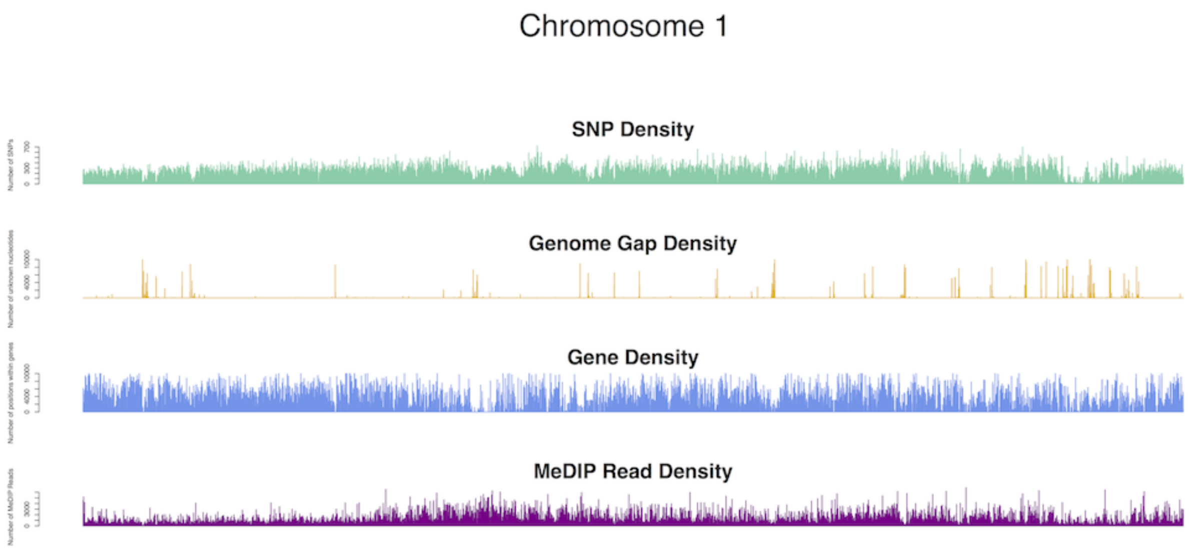

Figure S2: Feature profiles for chromosome 1.

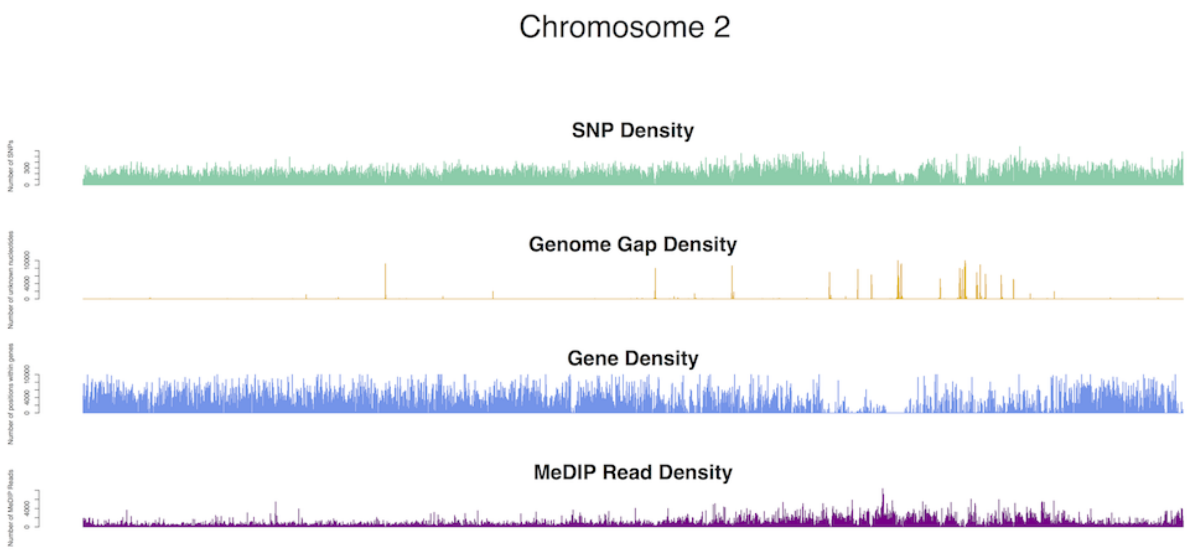

Figure S3: Feature profiles for chromosome 2.

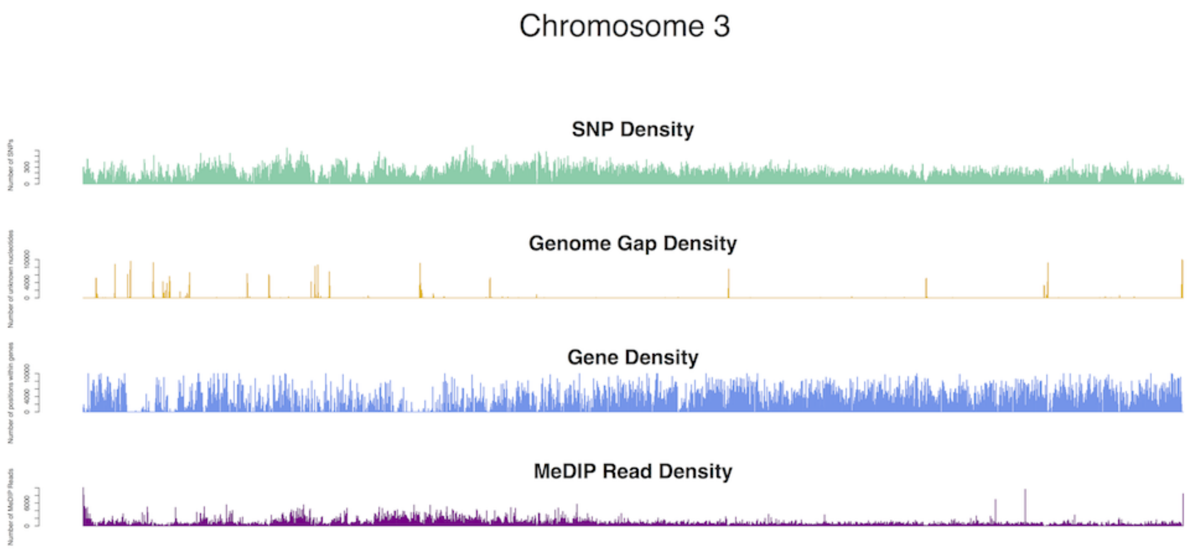

Figure S4: Feature profiles for chromosome 3.

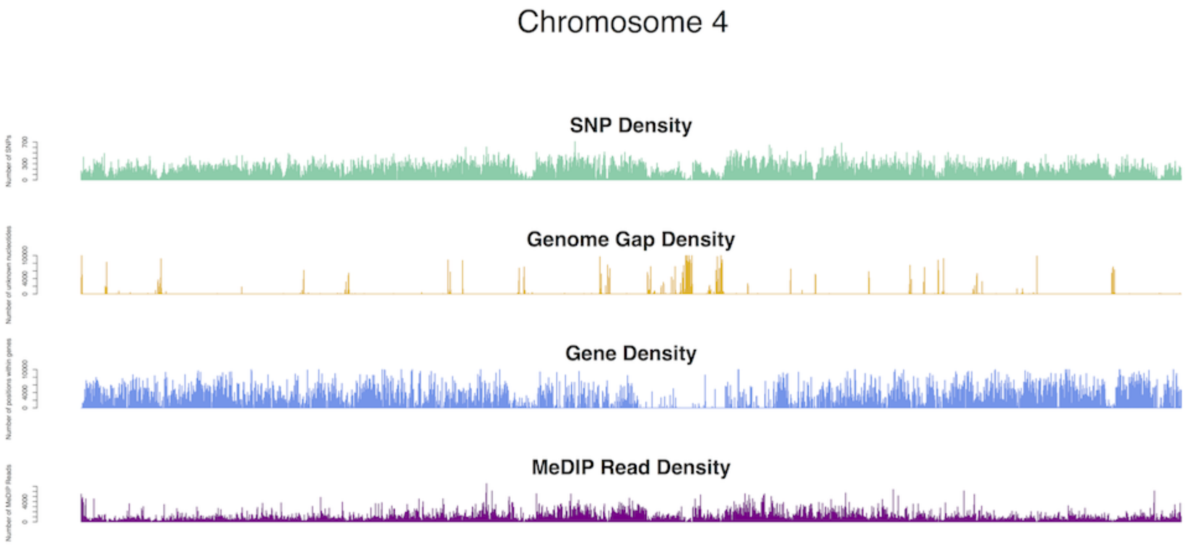

Figure S5: Feature profiles for chromosome 4.

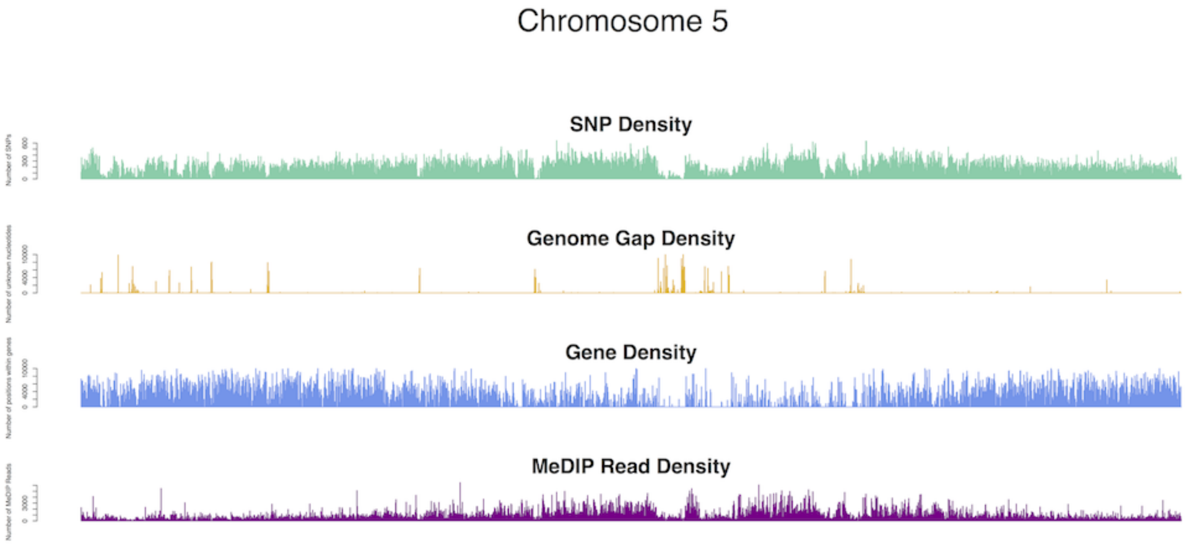

Figure S6: Feature profiles for chromosome 5.

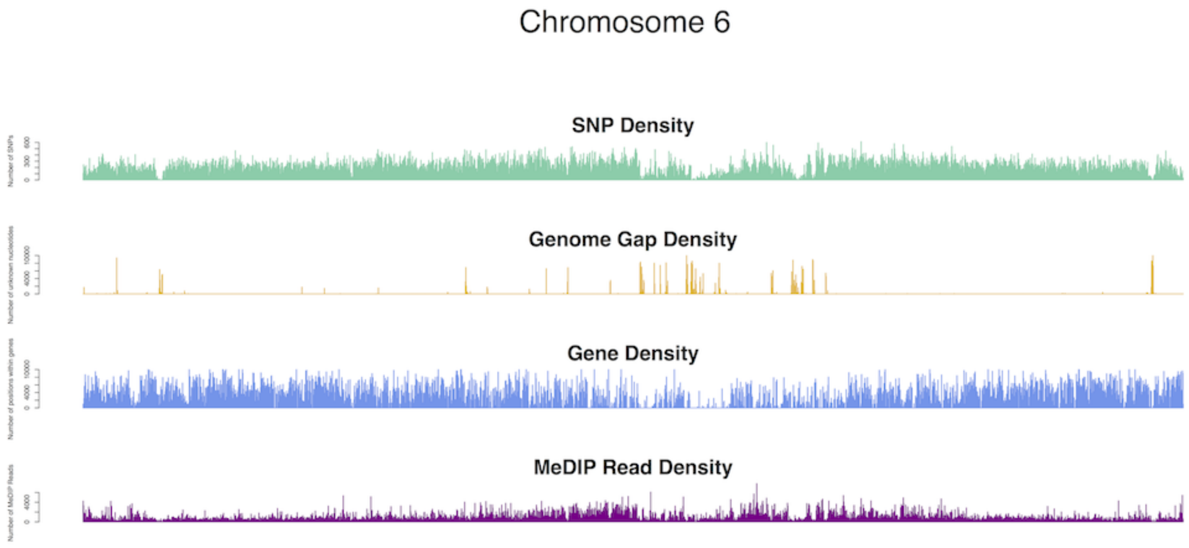

Figure S7: Feature profiles for chromosome 6.

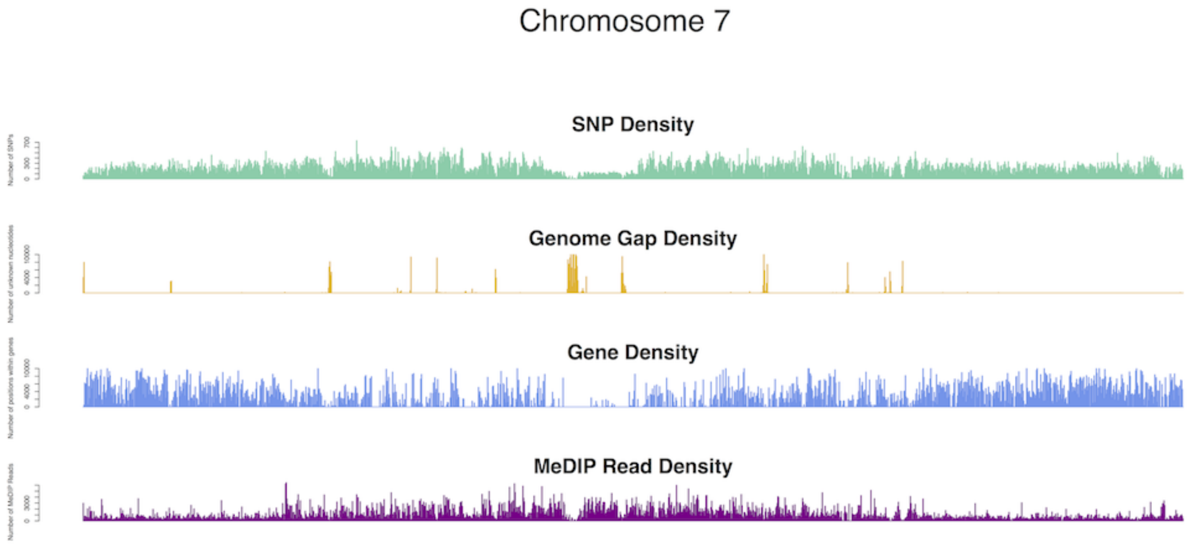

Figure S8: Feature profiles for chromosome 7.

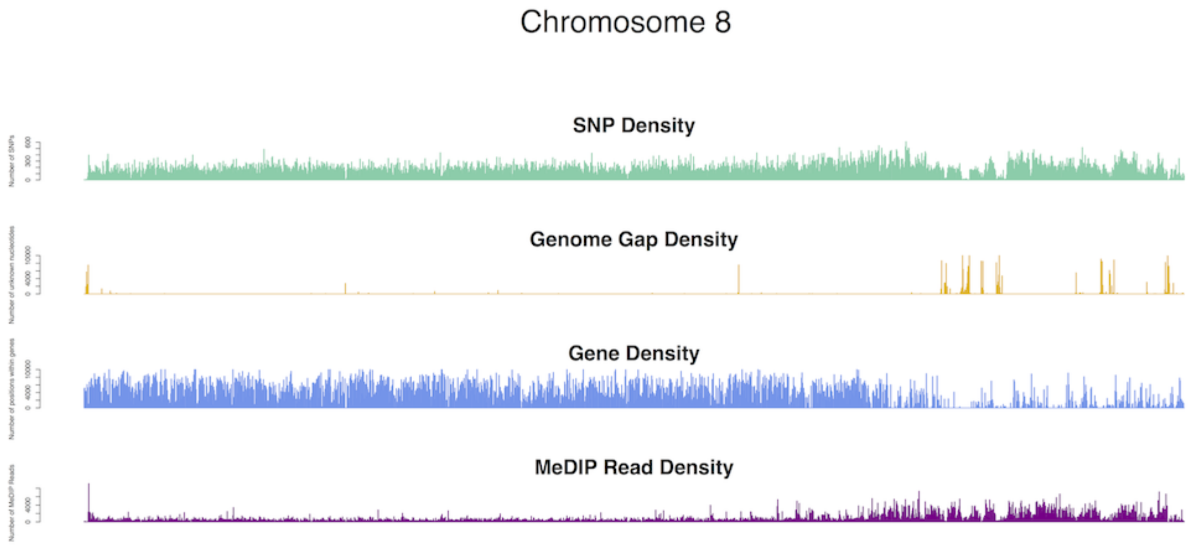

Figure S9: Feature profiles for chromosome 8.

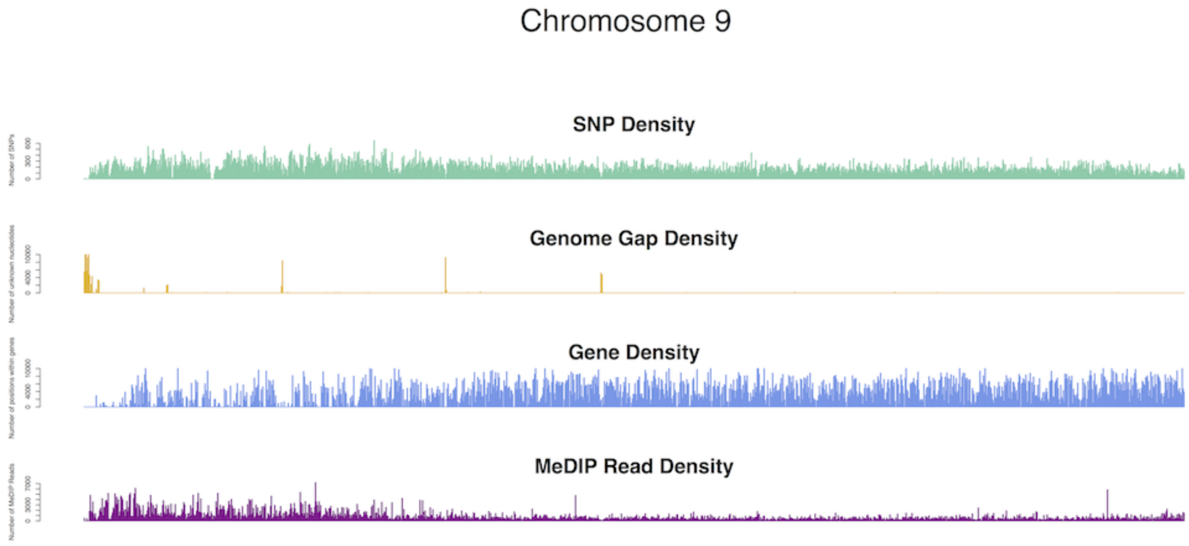

Figure S10: Feature profiles for chromosome 9.

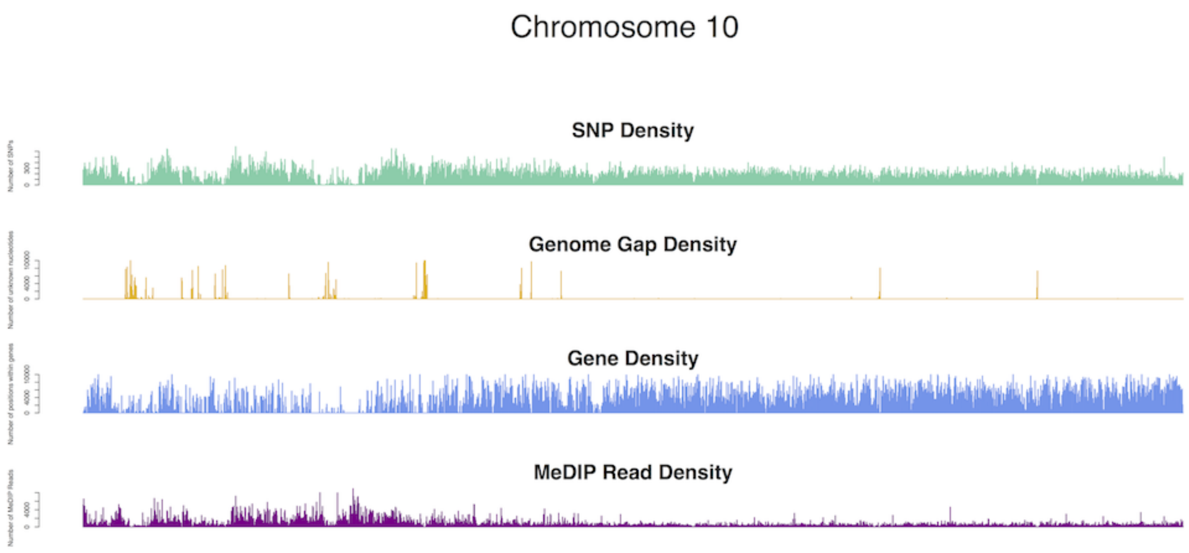

Figure S11: Feature profiles for chromosome 10.

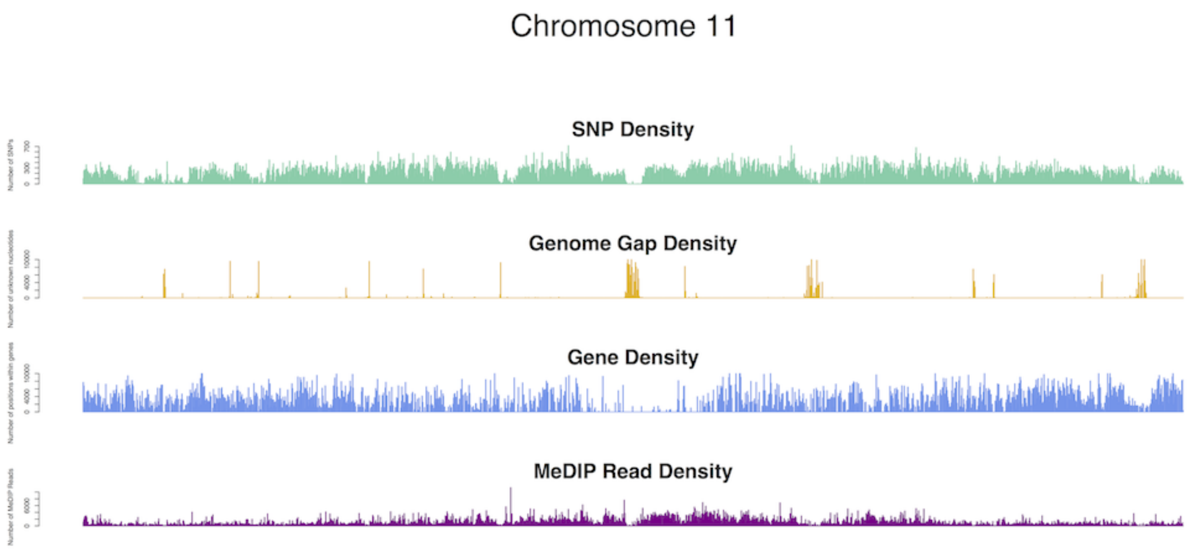

Figure S12: Feature profiles for chromosome 11.

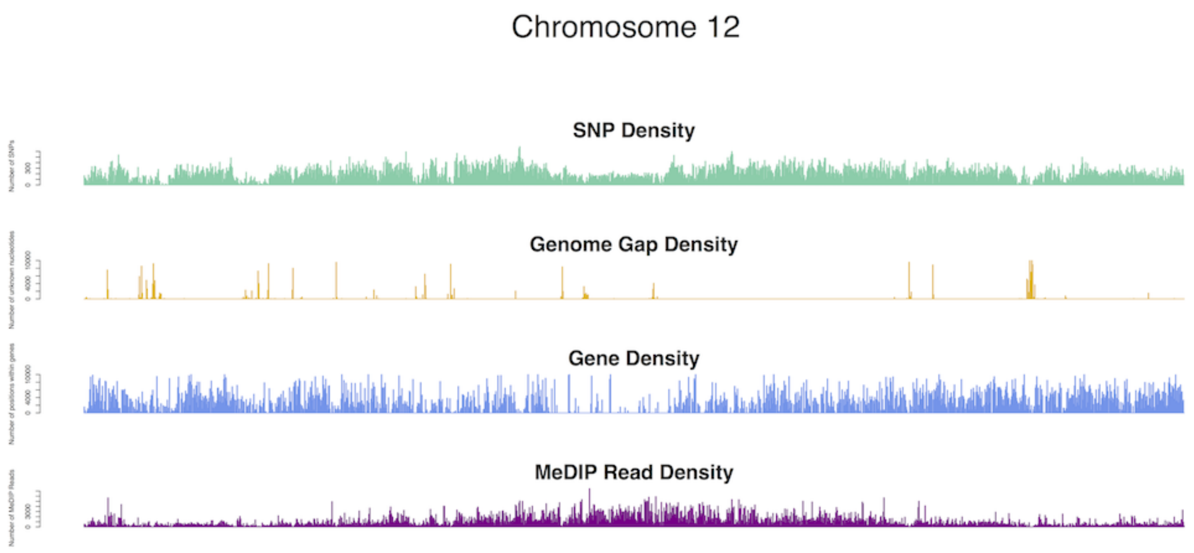

Figure S13: Feature profiles for chromosome 12.

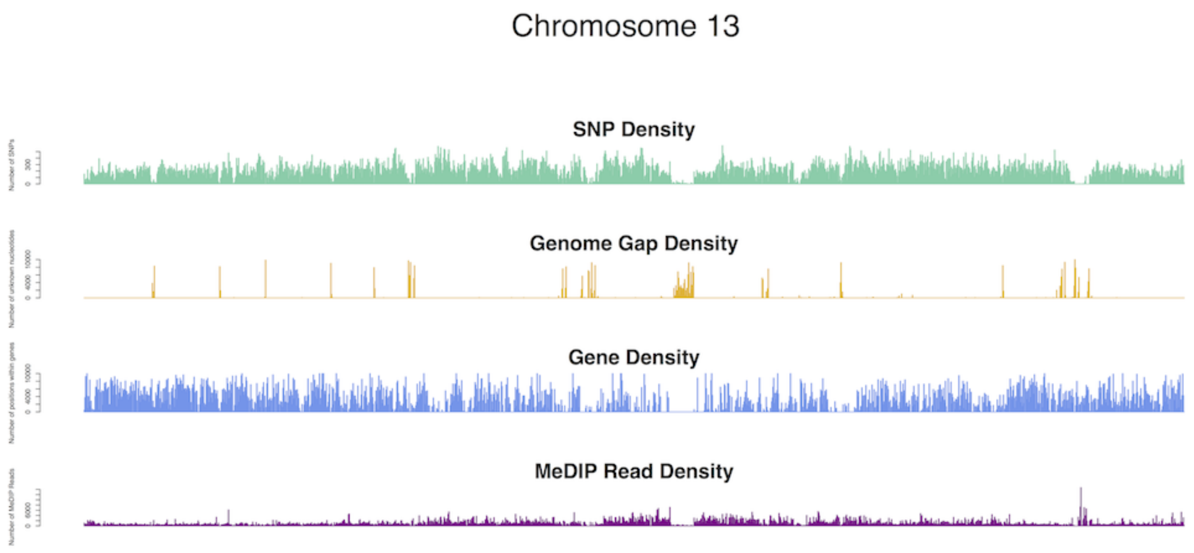

Figure S14: Feature profiles for chromosome 13.

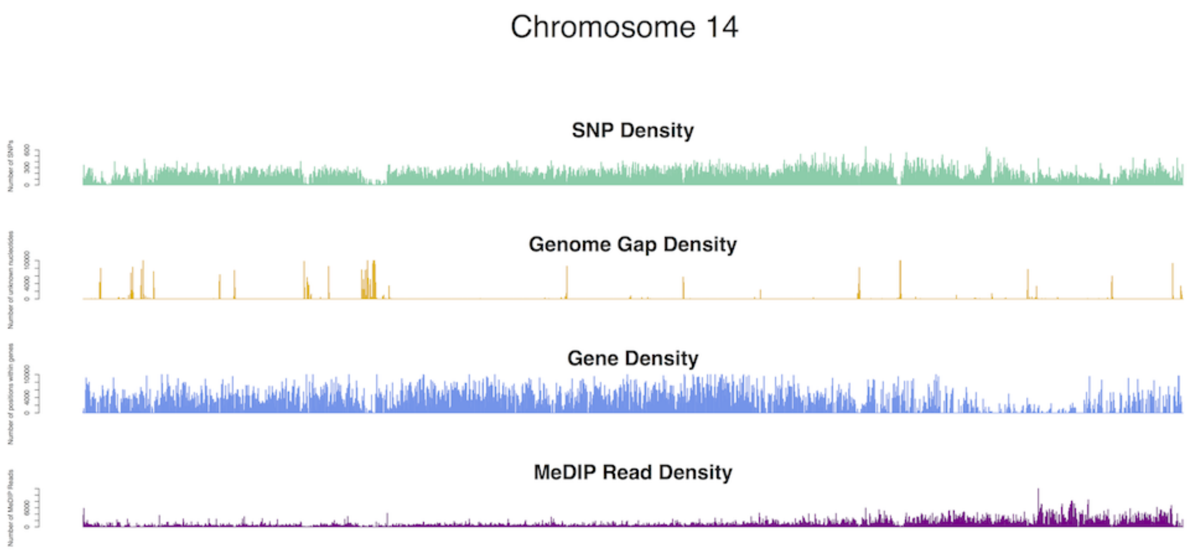

Figure S15: Feature profiles for chromosome 14.

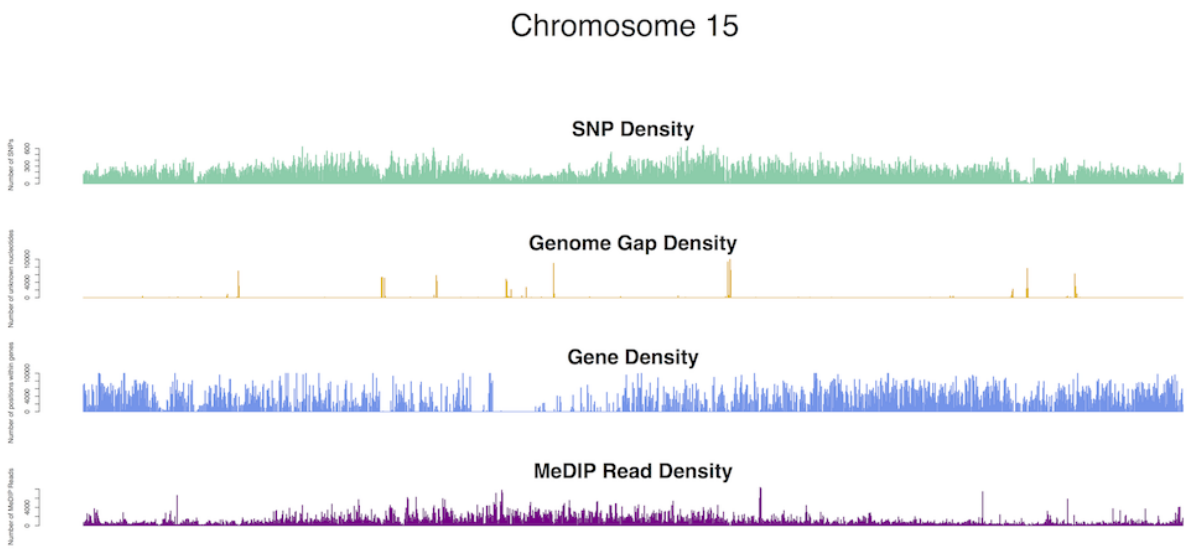

Figure S16: Feature profiles for chromosome 15.

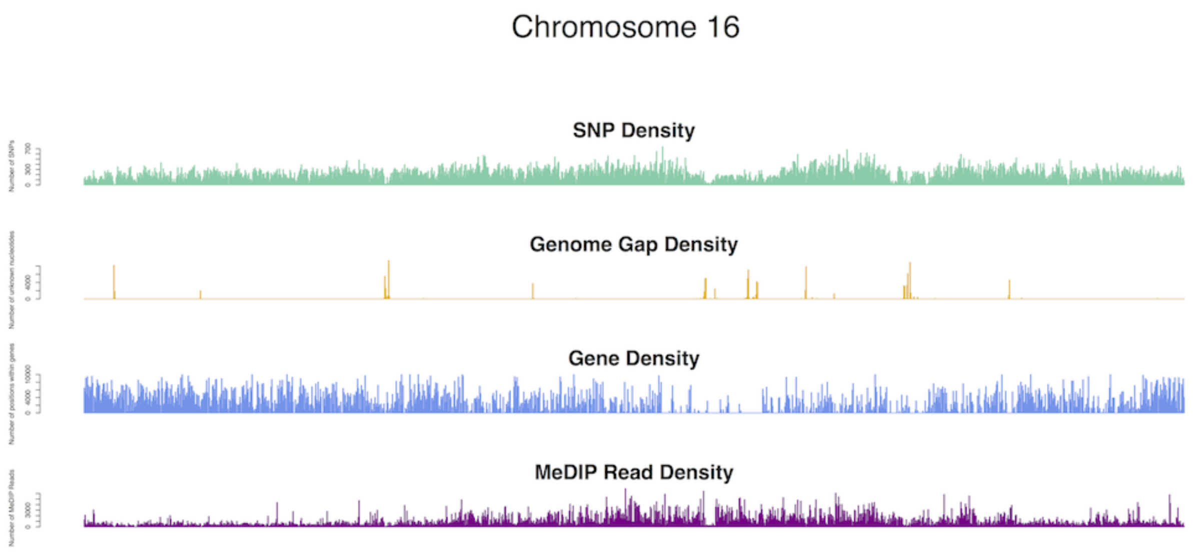

Figure S17: Feature profiles for chromosome 16.

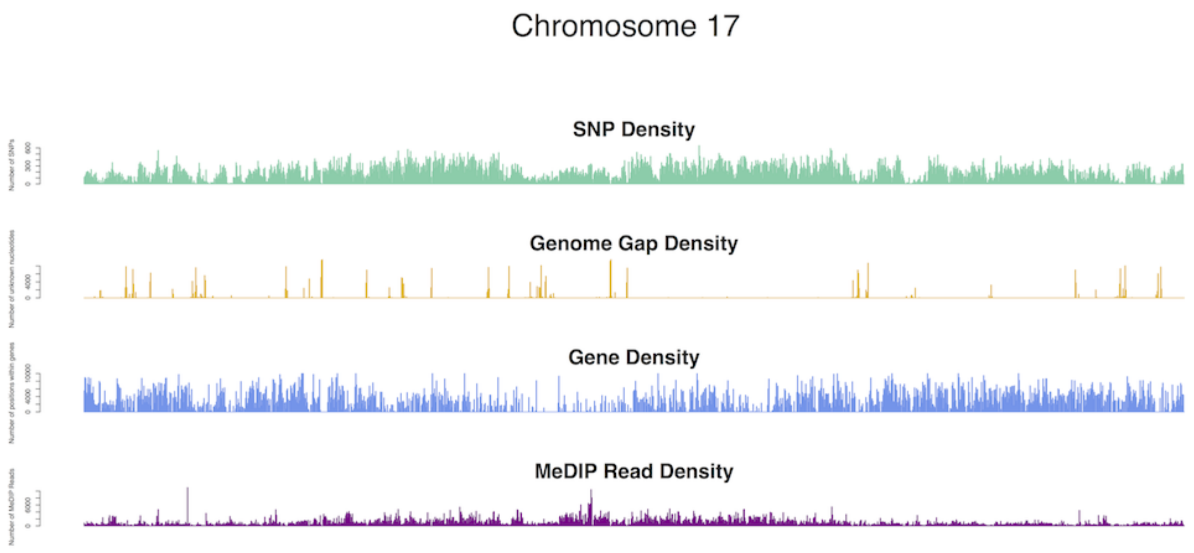

Figure S18: Feature profiles for chromosome 17.

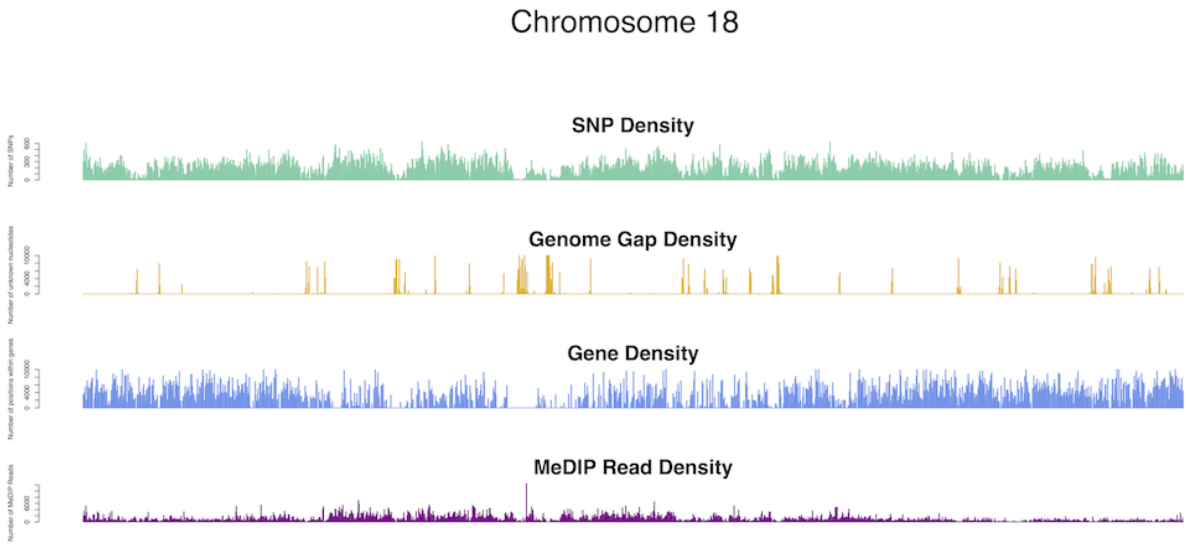

Figure S19: Feature profiles for chromosome 18.

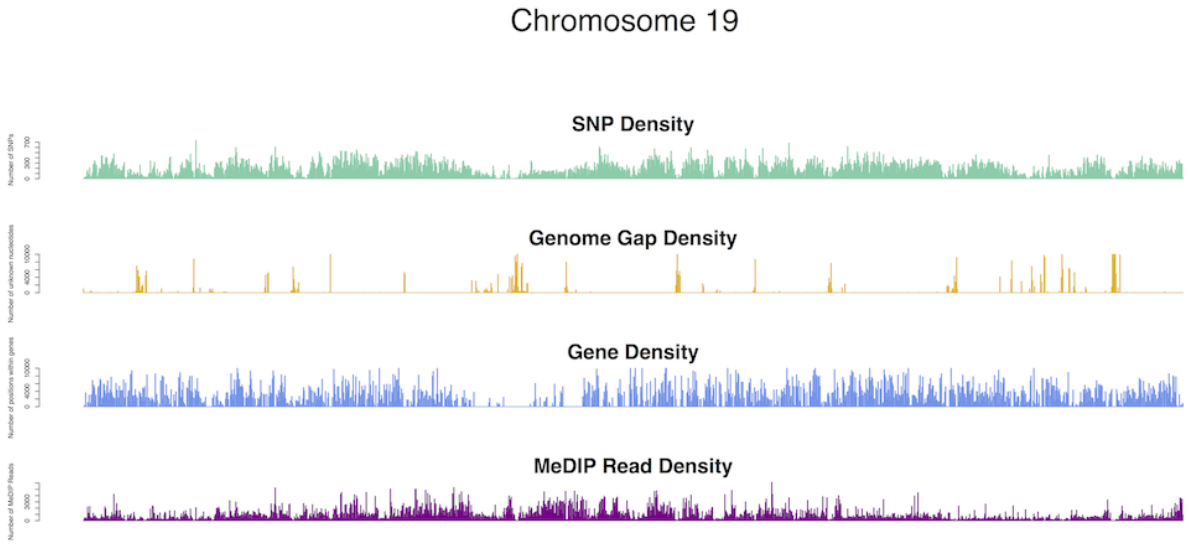

Figure S20: Feature profiles for chromosome 19.

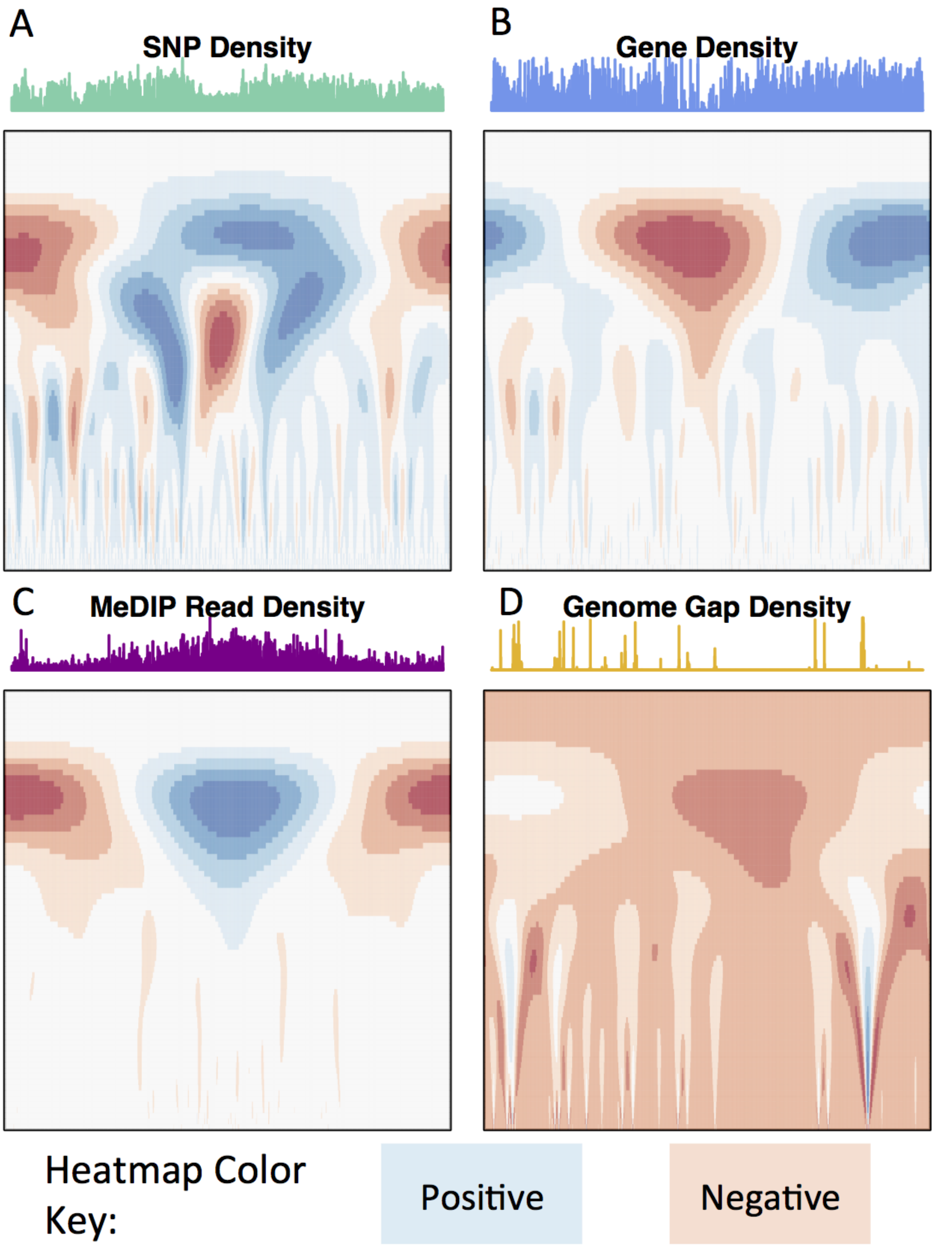

Figure S21: CWT Coefficient landscapes of chromosome 12 for (A) SNP density, (B) gene density, (C) methylation (MeDIP-Seq read density, internode explant tissue) and (D) genome gap density.

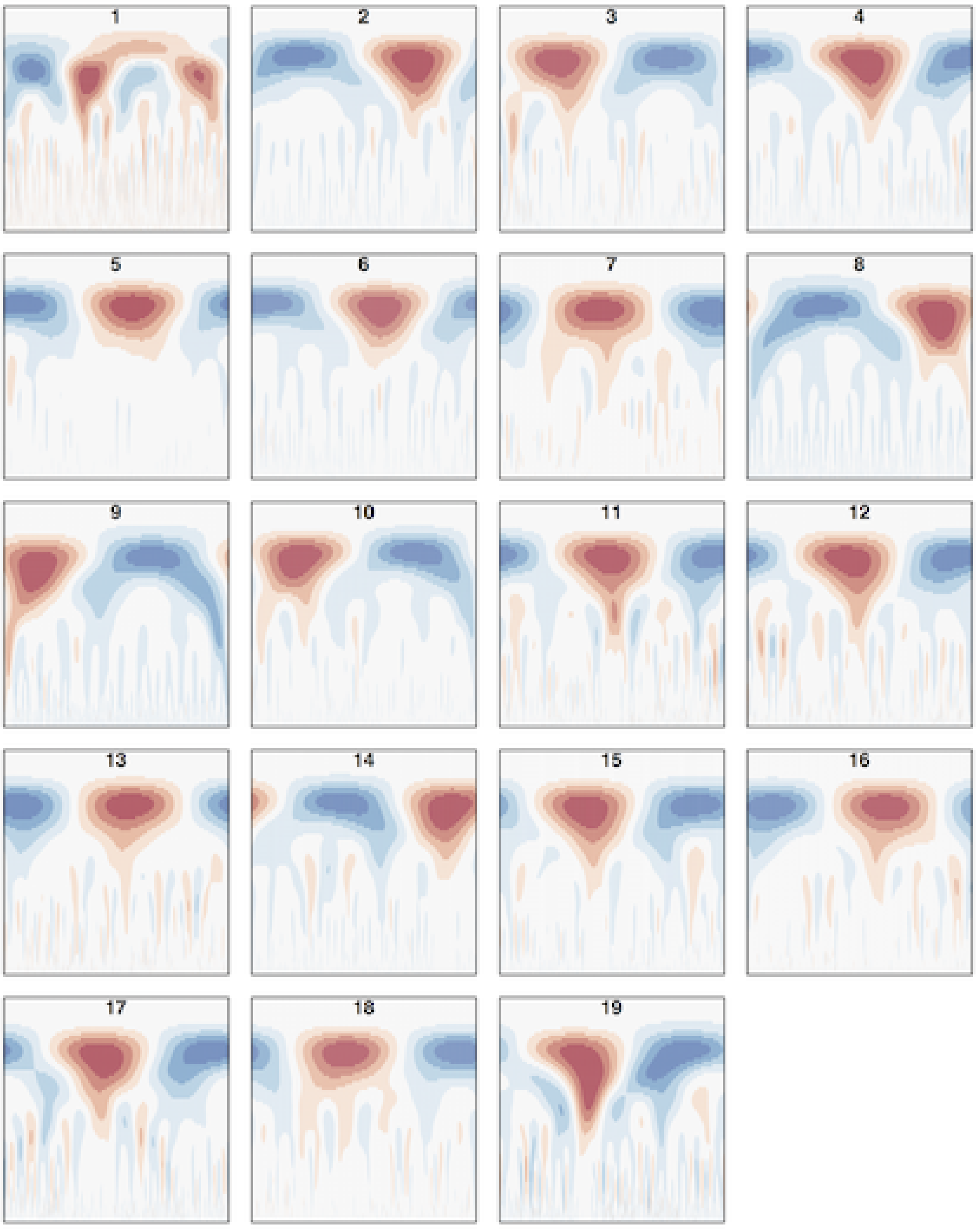

Figure S22: CWT landscape for gene density.

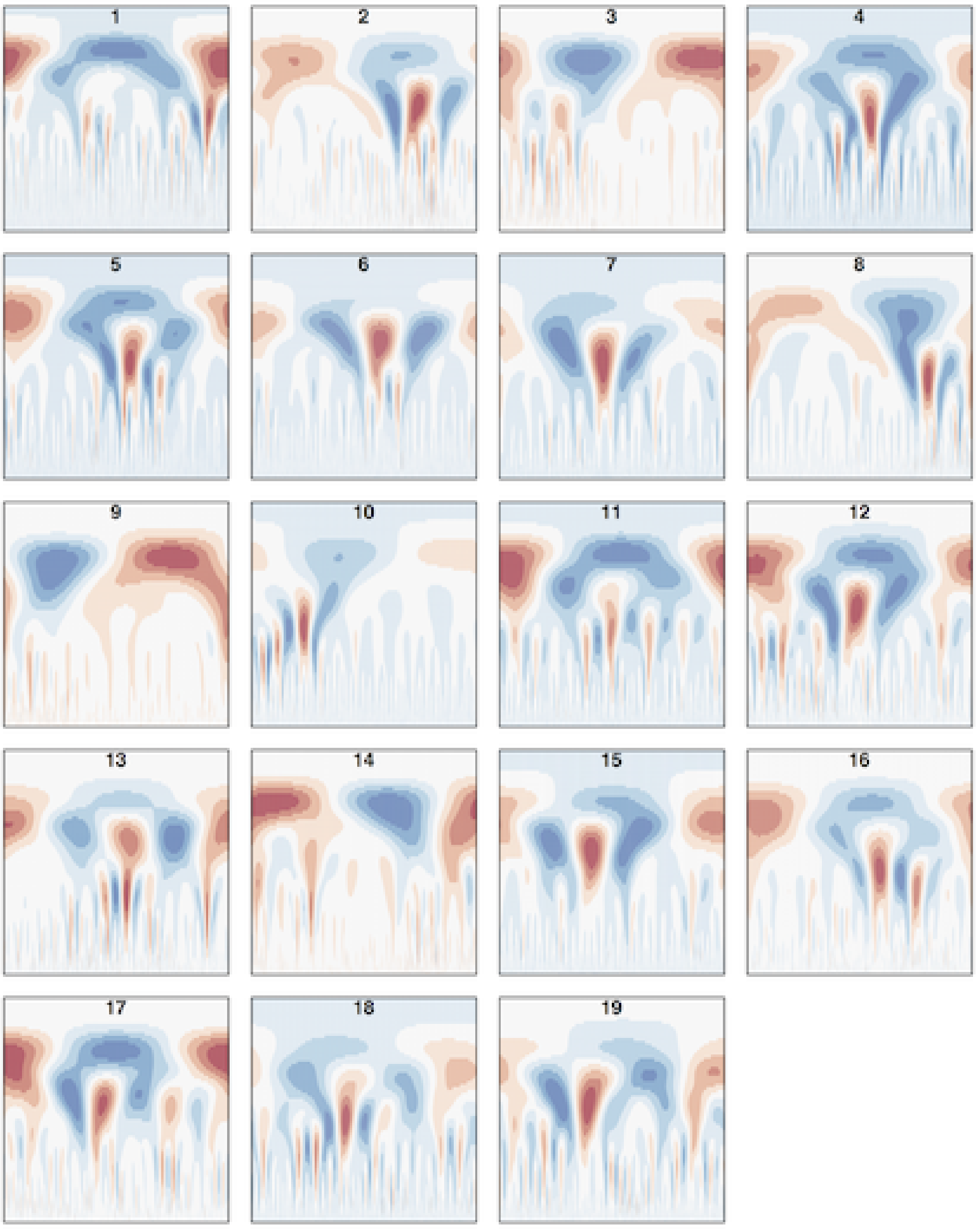

Figure S23: CWT landscape for SNP density.

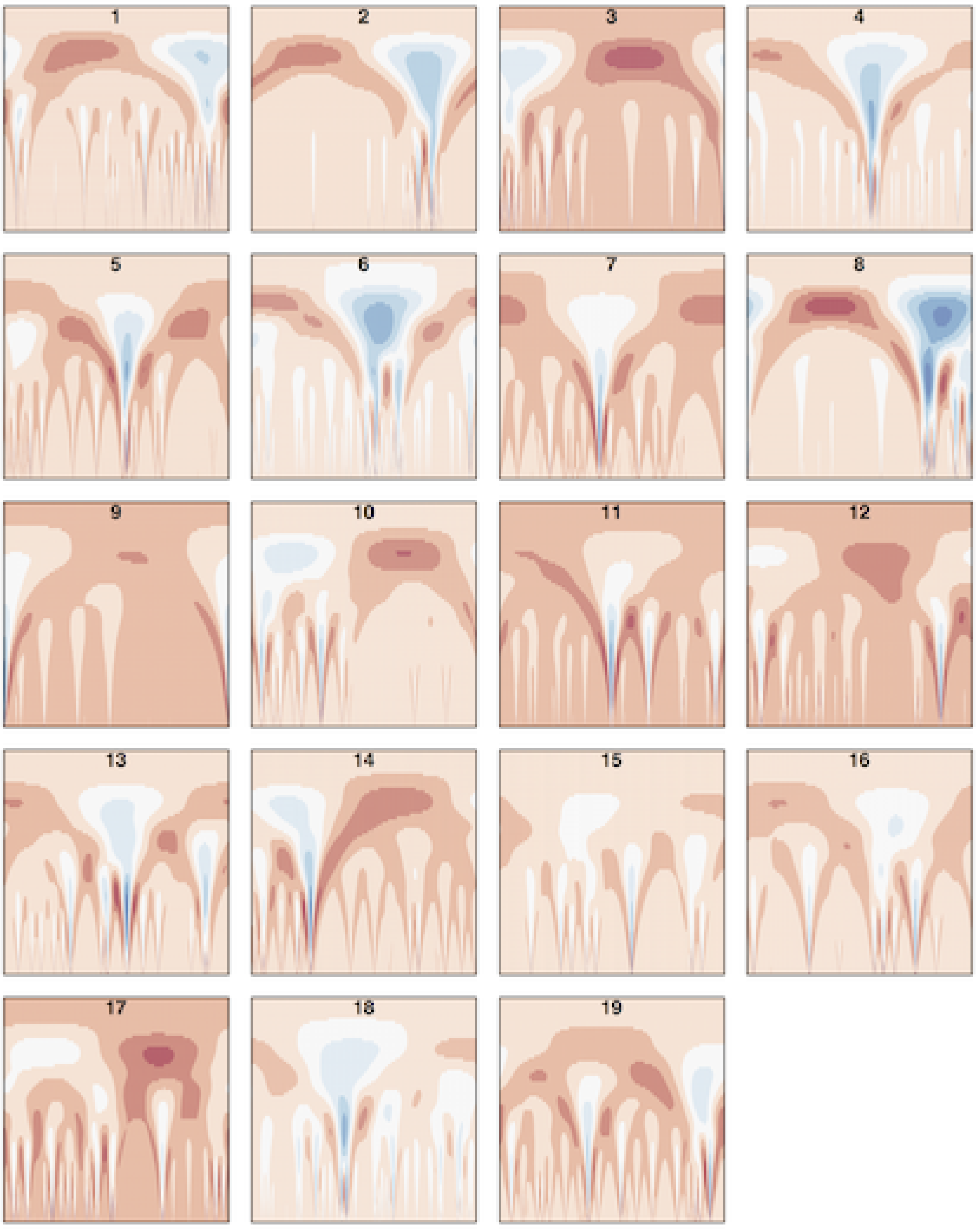

Figure S24: CWT landscape for genome gap density.

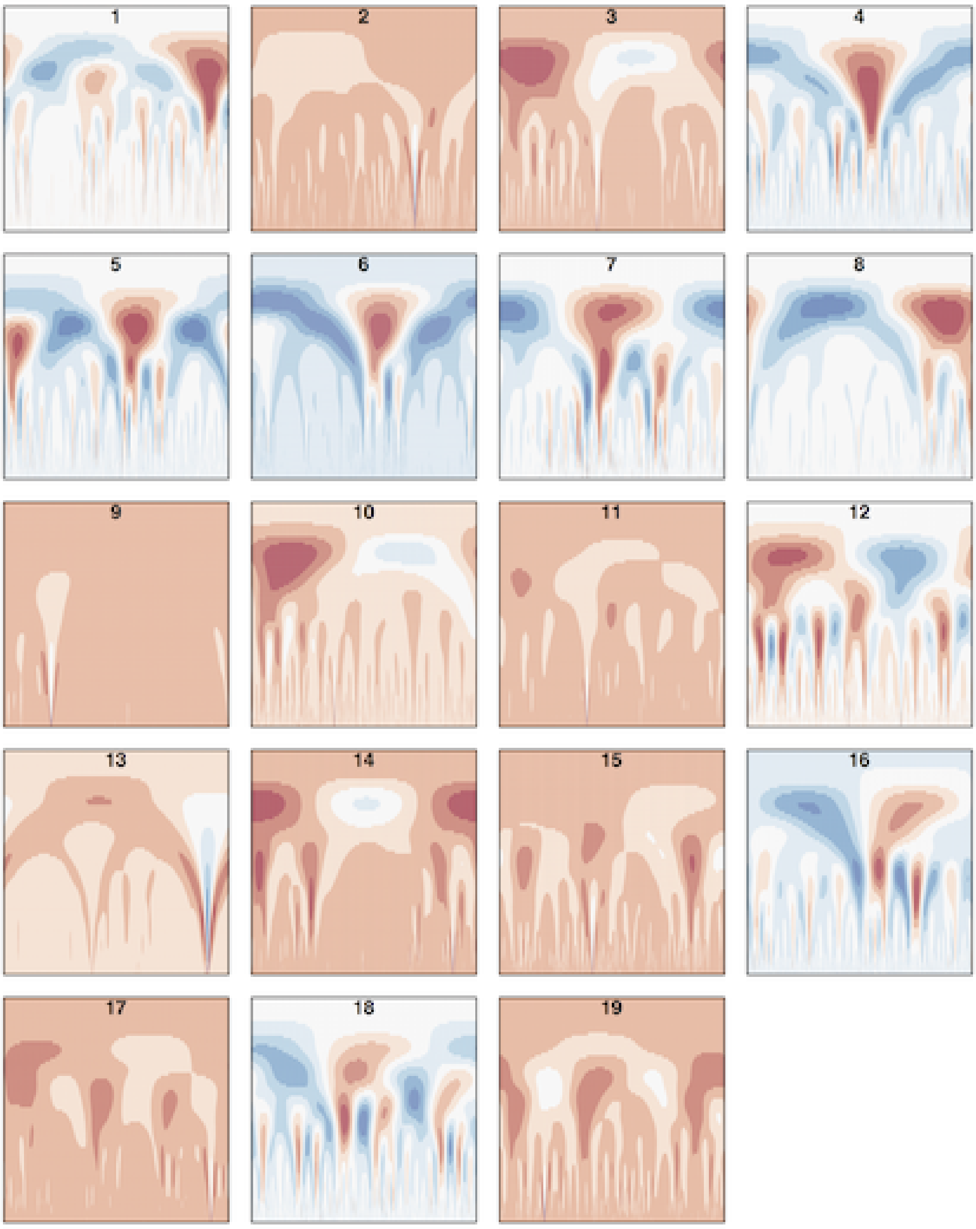

Figure S25: CWT methylation landscape for bud tissue.

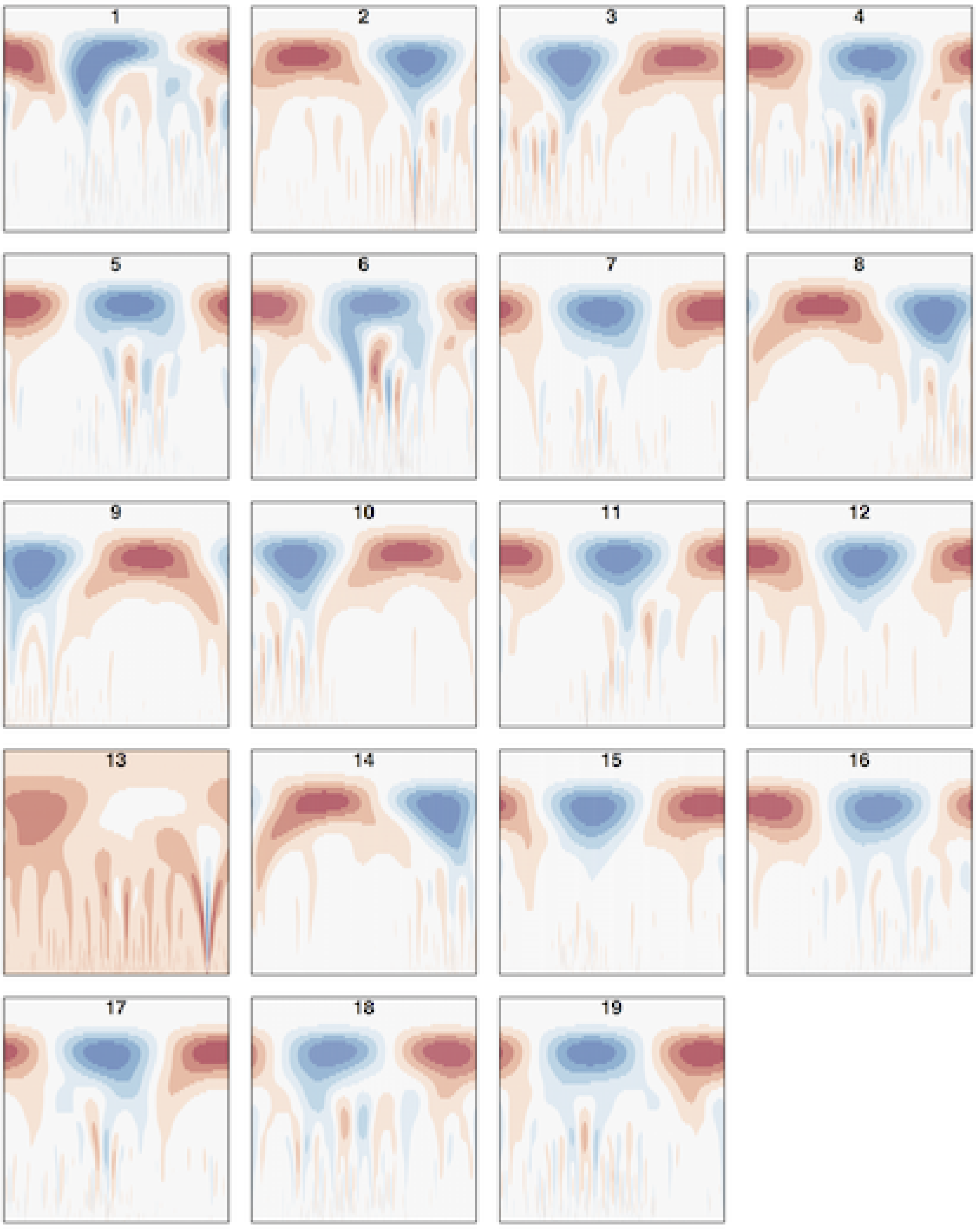

Figure S26: CWT methylation landscape for callus tissue.

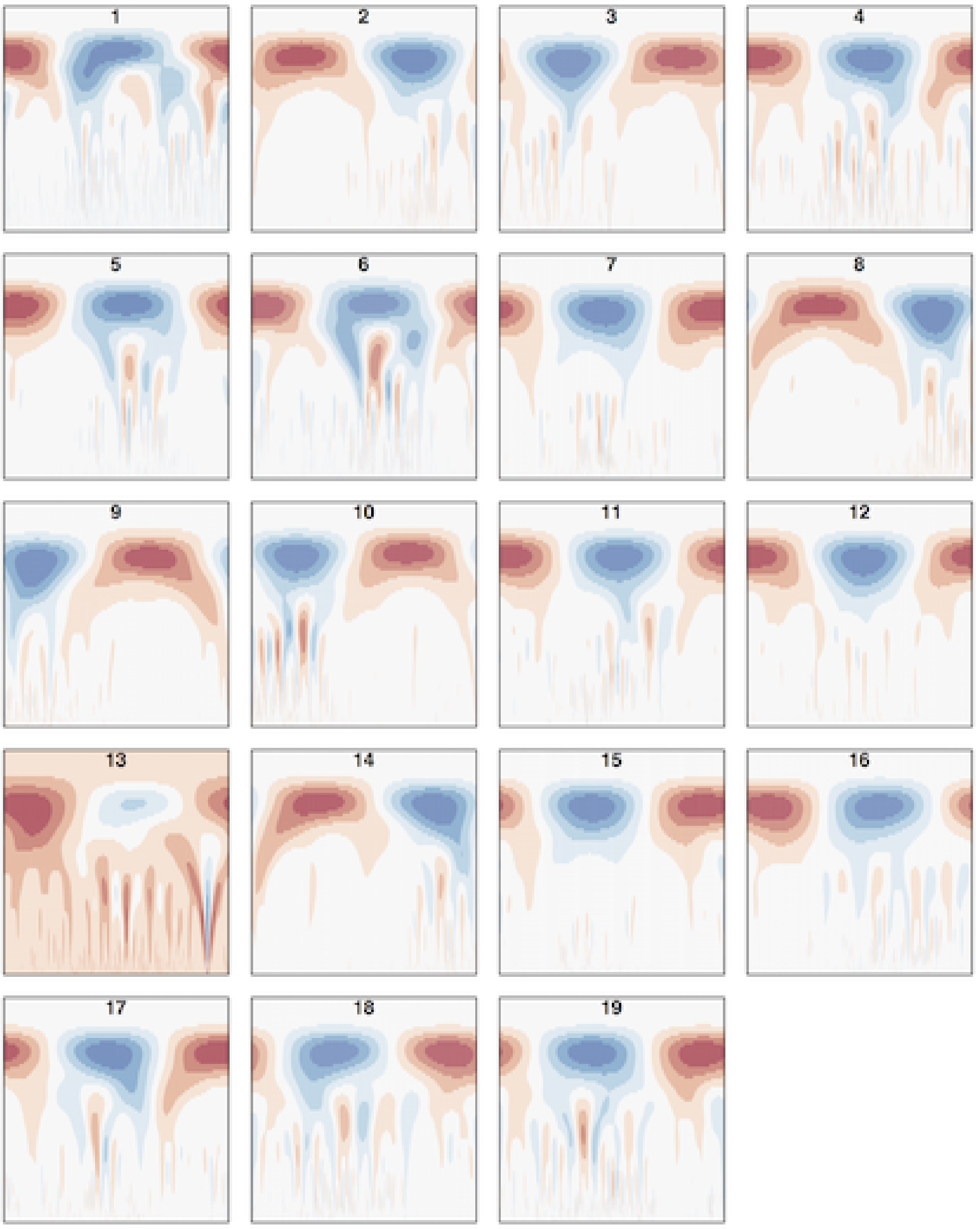

Figure S27: CWT methylation landscape for female catkin tissue.

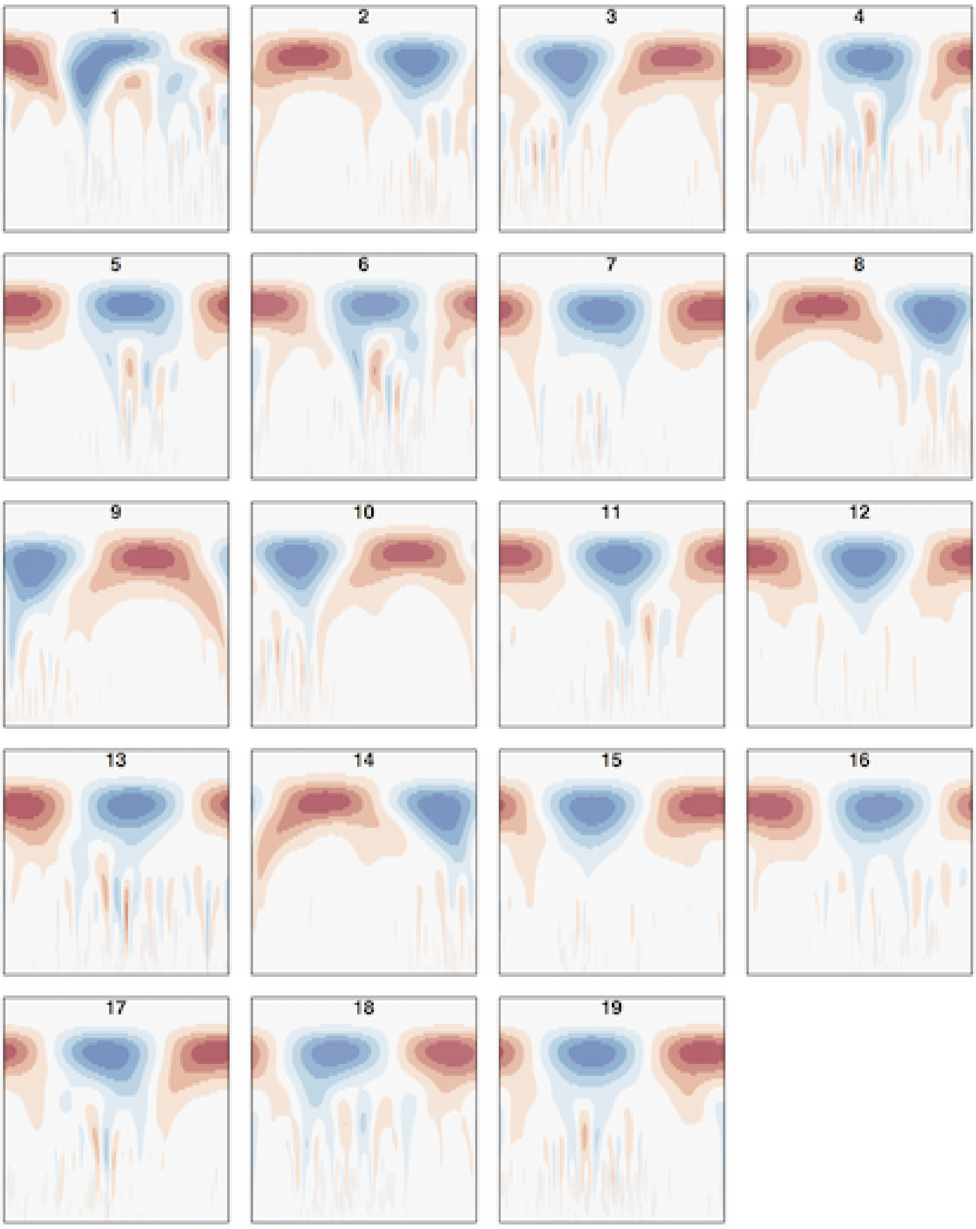

Figure S28: CWT methylation landscape for internode explant tissue.

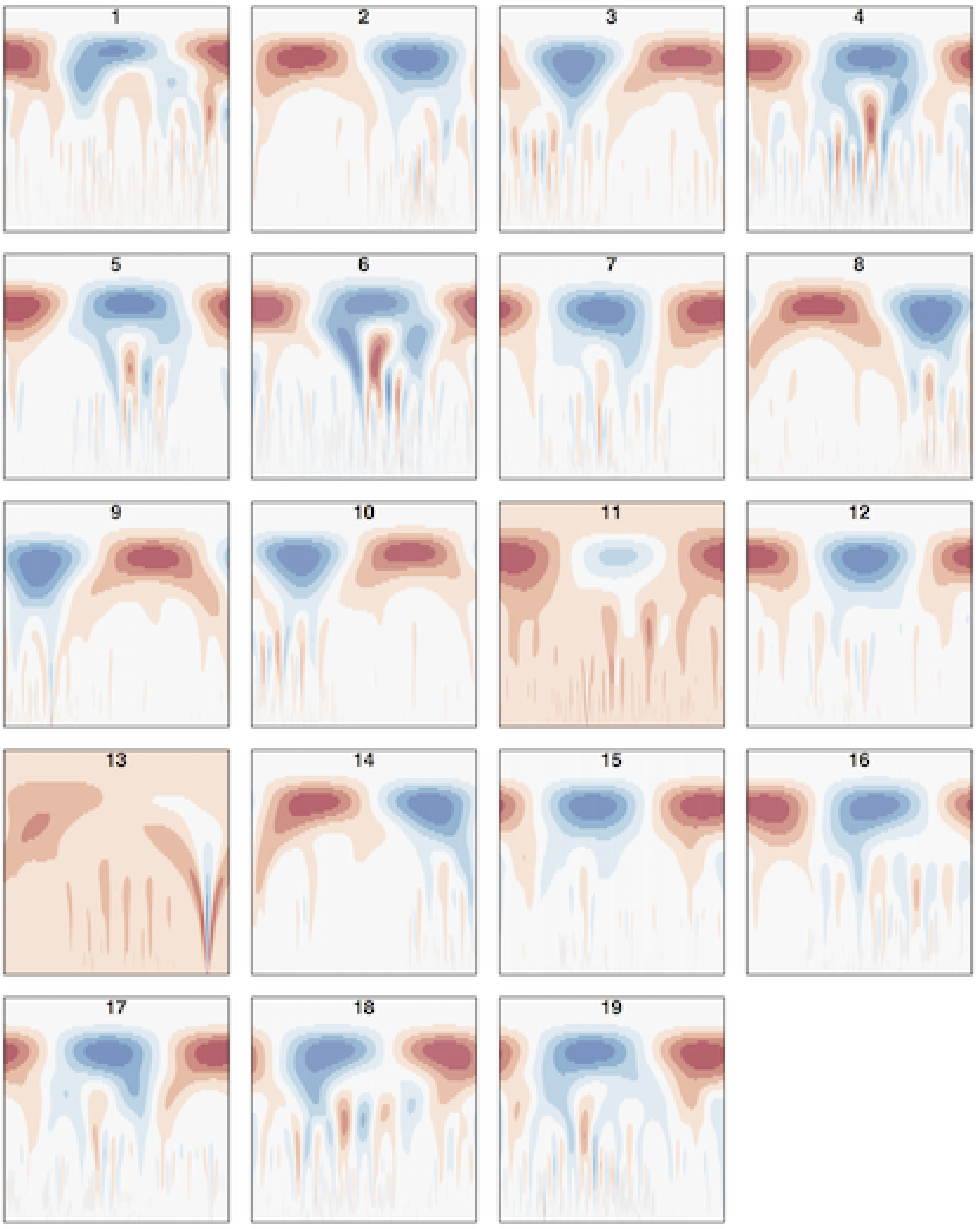

Figure S29: CWT methylation landscape for leaf tissue.

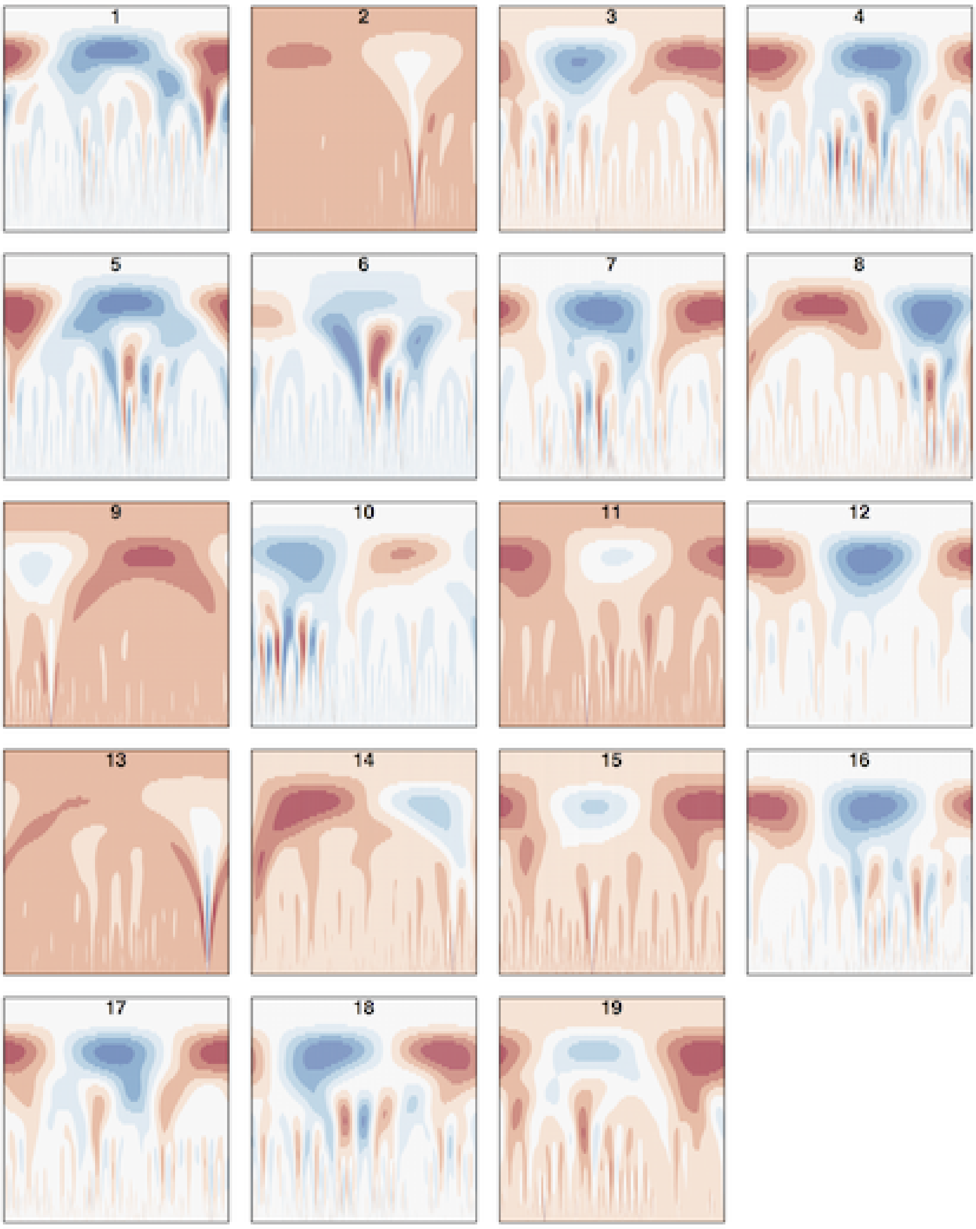

Figure S30: CWT methylation landscape for male catkin tissue.

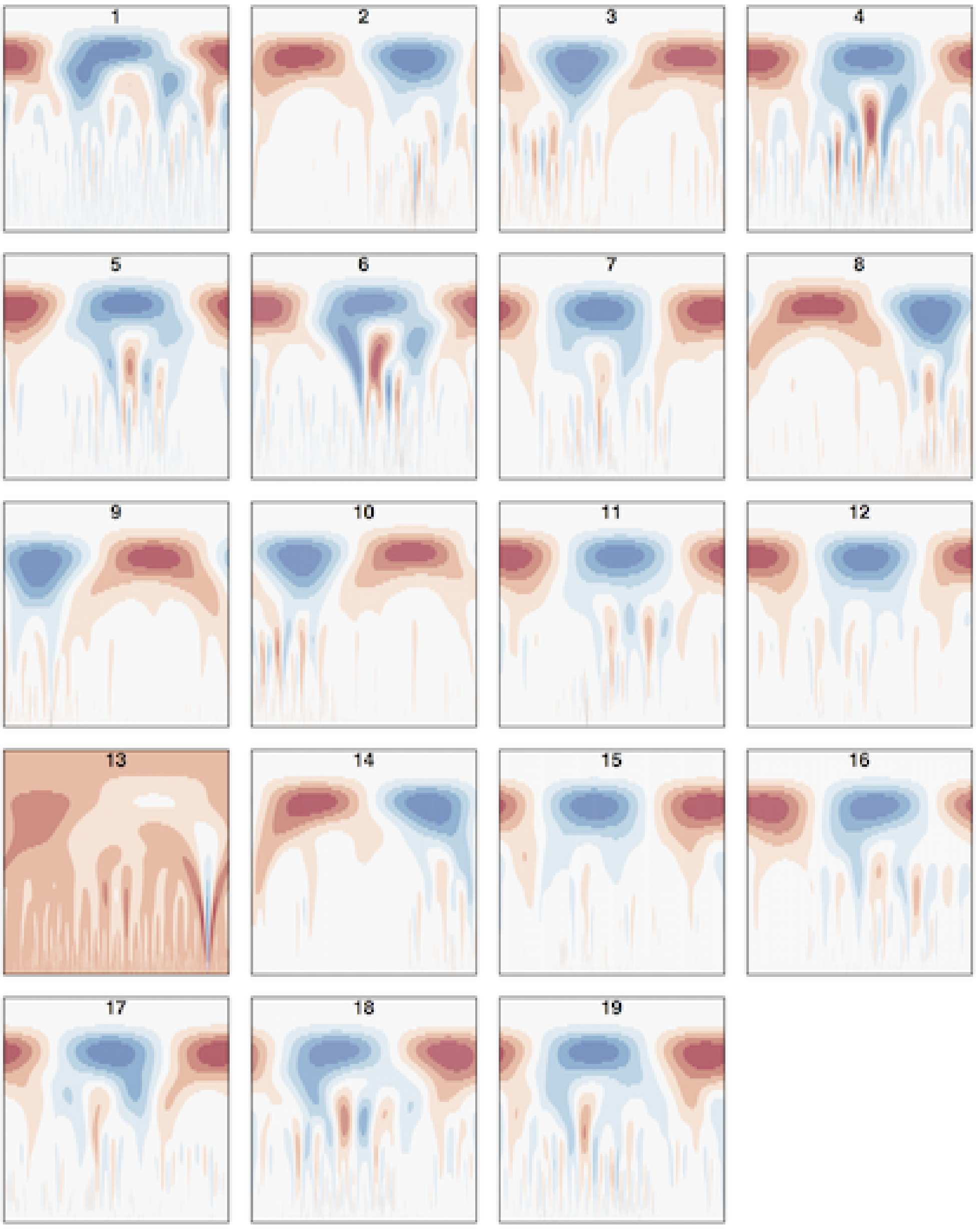

Figure S31: CWT methylation landscape for phloem tissue.

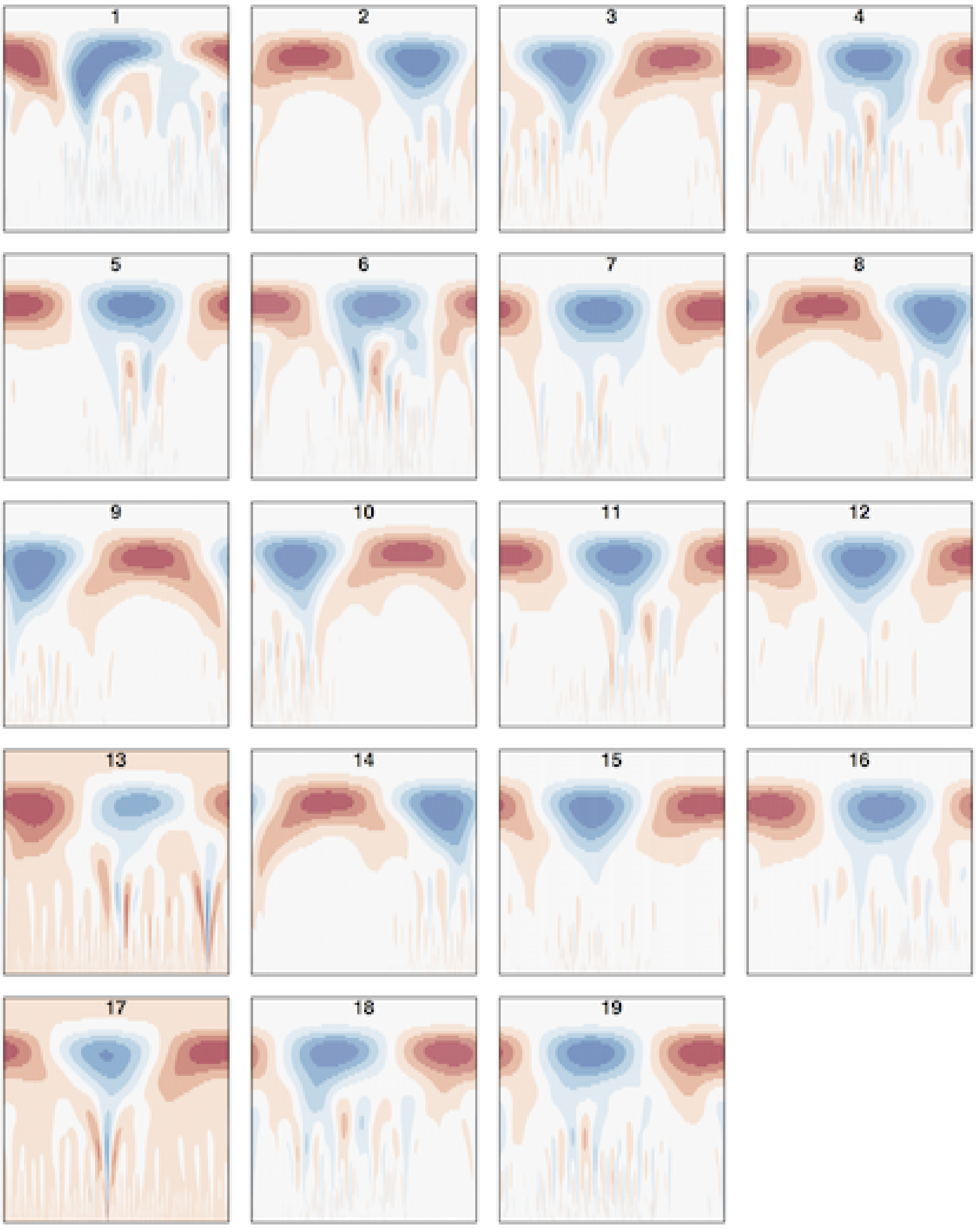

Figure S32: CWT methylation landscape for regenerated internode tissue.

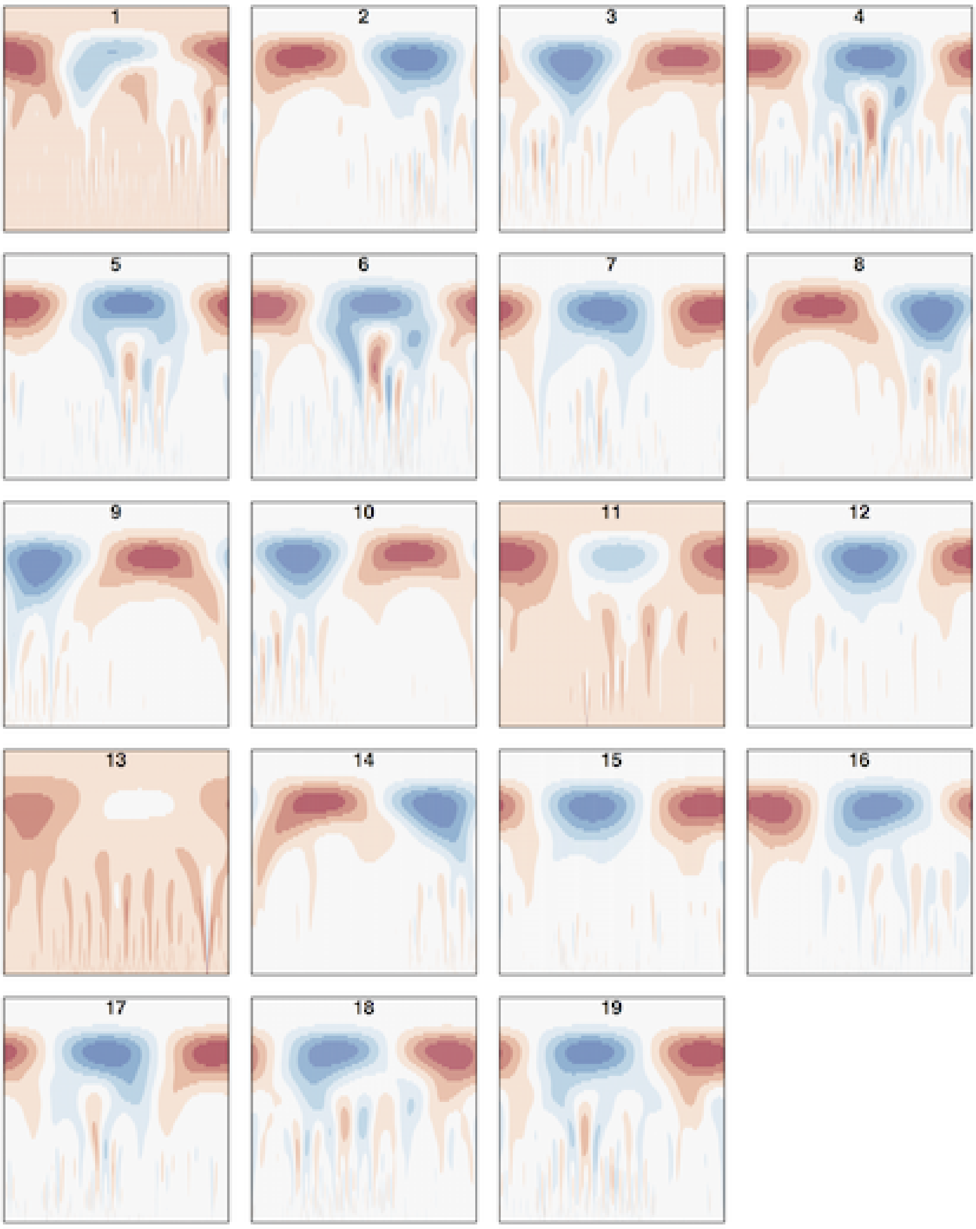

Figure S33: CWT methylation landscape for root tissue.

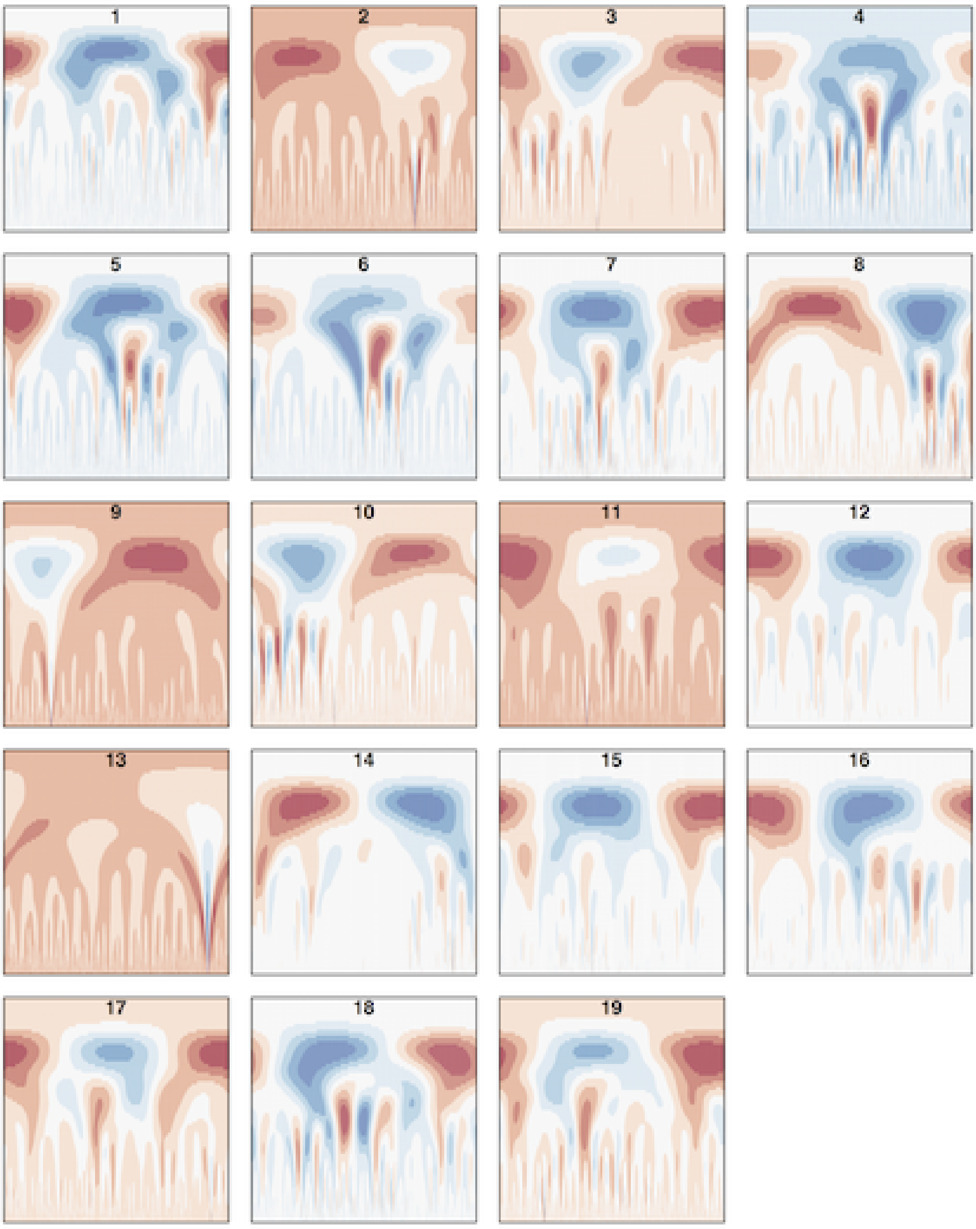

Figure S34: CWT landscape for xylem tissue.

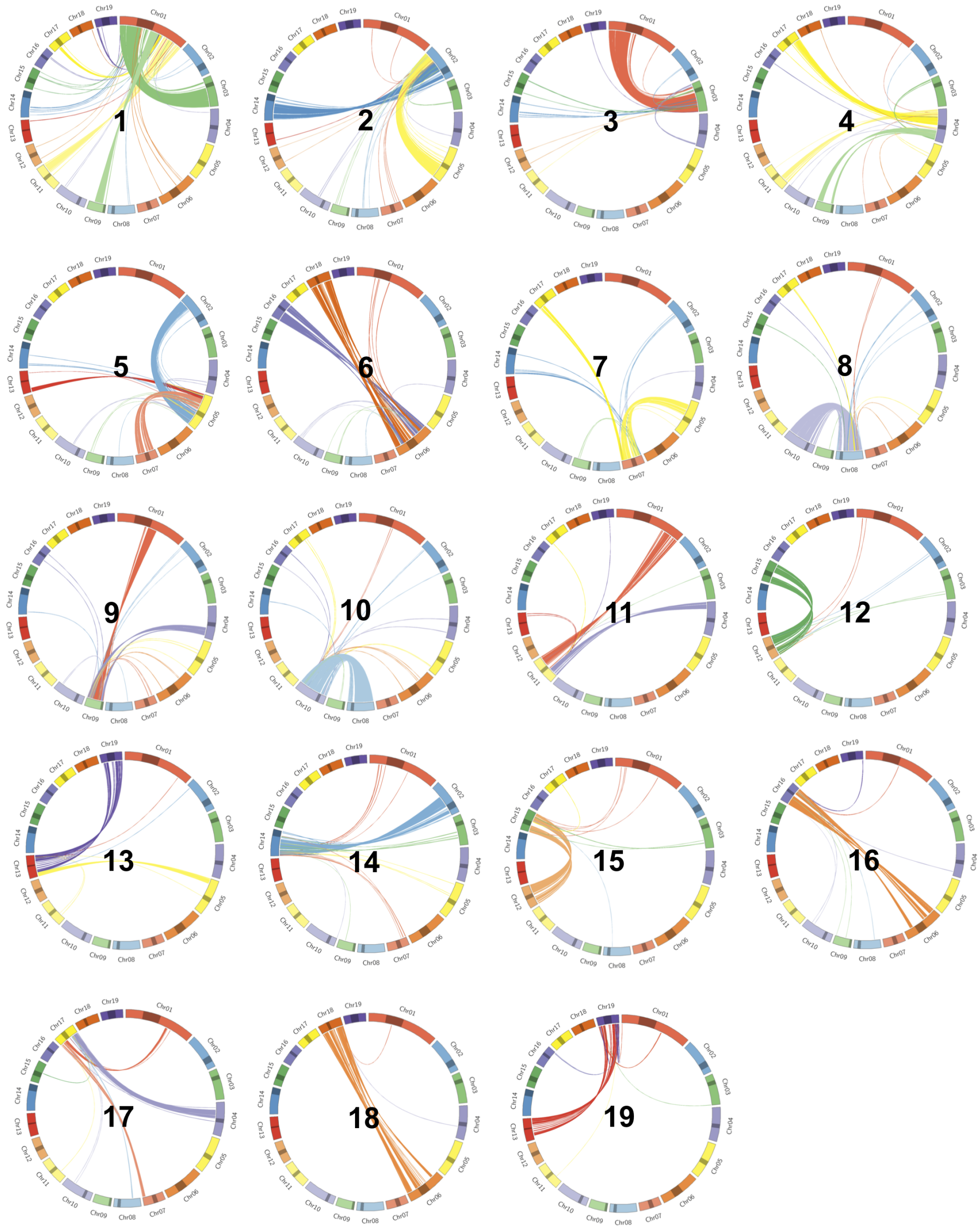

Figure S35: Circos plots representing syntenic blocks, centered around each chromosome, individually. Chromosome lengths are represented on the ideograms with the predicted centromeric/pericentromeric regions shown as dark highlights. Links between chromosomes indicate homologous chromosome regions, and are colored according to the source chromosome. The target chromosome is indicated by the number within each circos plot.

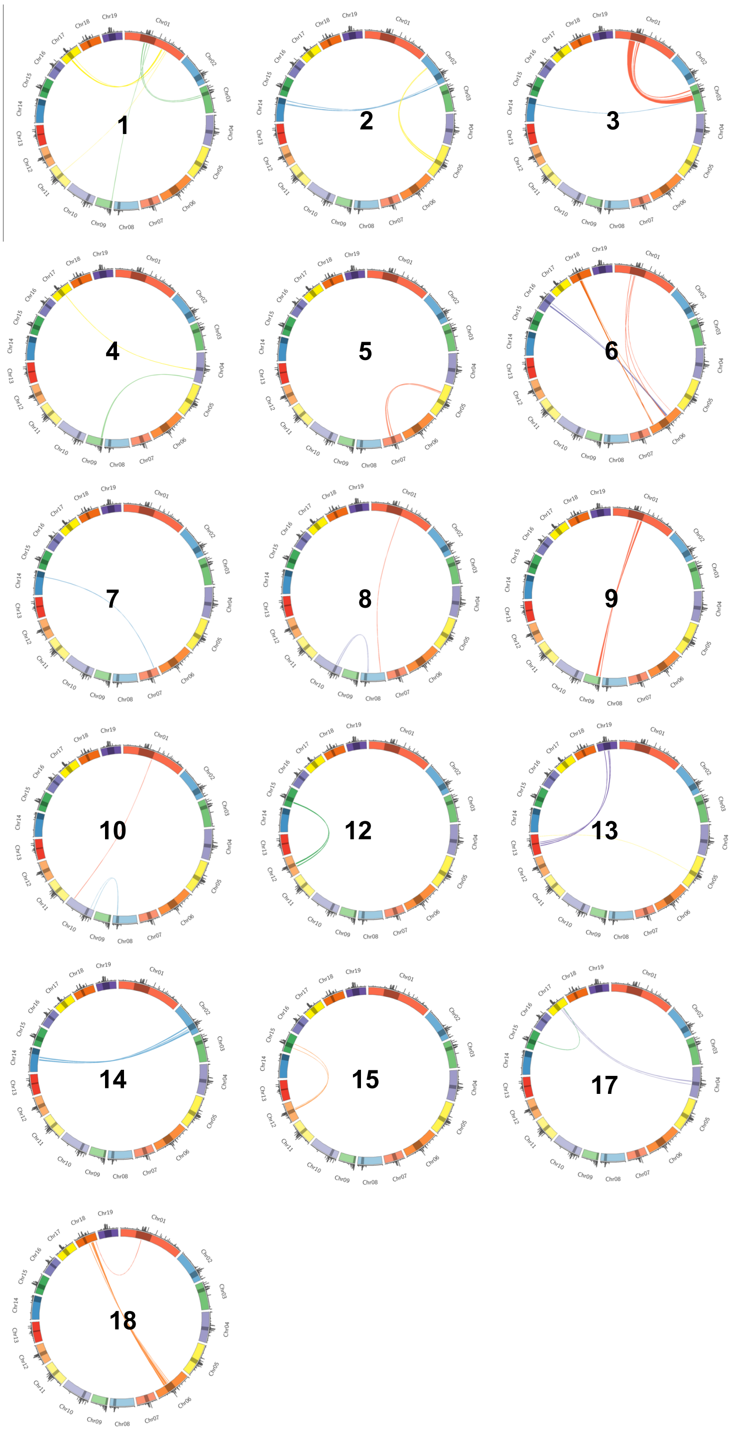

Figure S36: Syntenic blocks from Figure S35 which overlap with predicted centromeric/pericentromeric regions on source chromosomes. PGSB centromere repeat densities from Figure 9 in the main text are included as a bar chart on the ideogram.

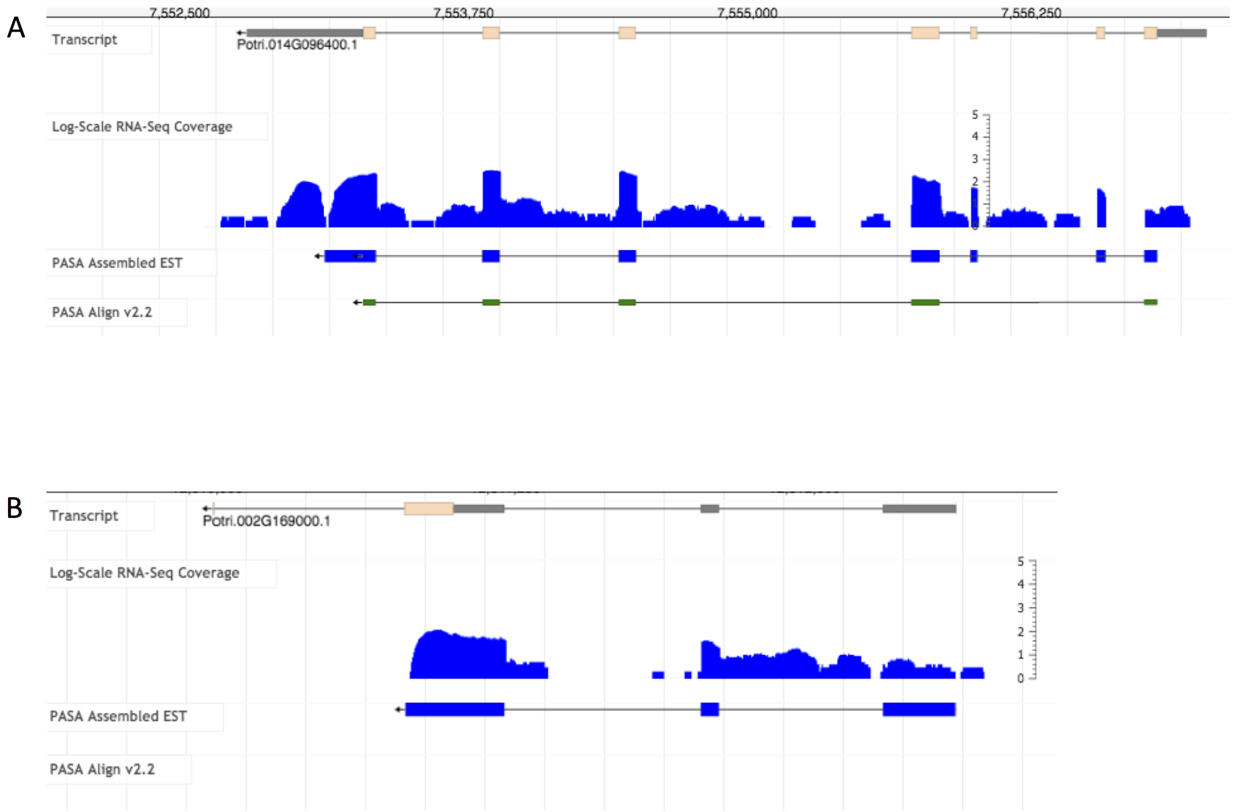

Figure S37: CENH3 candidate genes expression evidence. Two CENH3 candidates in *P. trichocarpa* (A) Potri.014G096400 and (B) Potri.002G169000 both show evidence of expression in the RNA-seq and EST coverage. Figure obtained using the Jbrowse plugin Skinner et al. (2009) on Phytozome Goodstein et al. (2012).

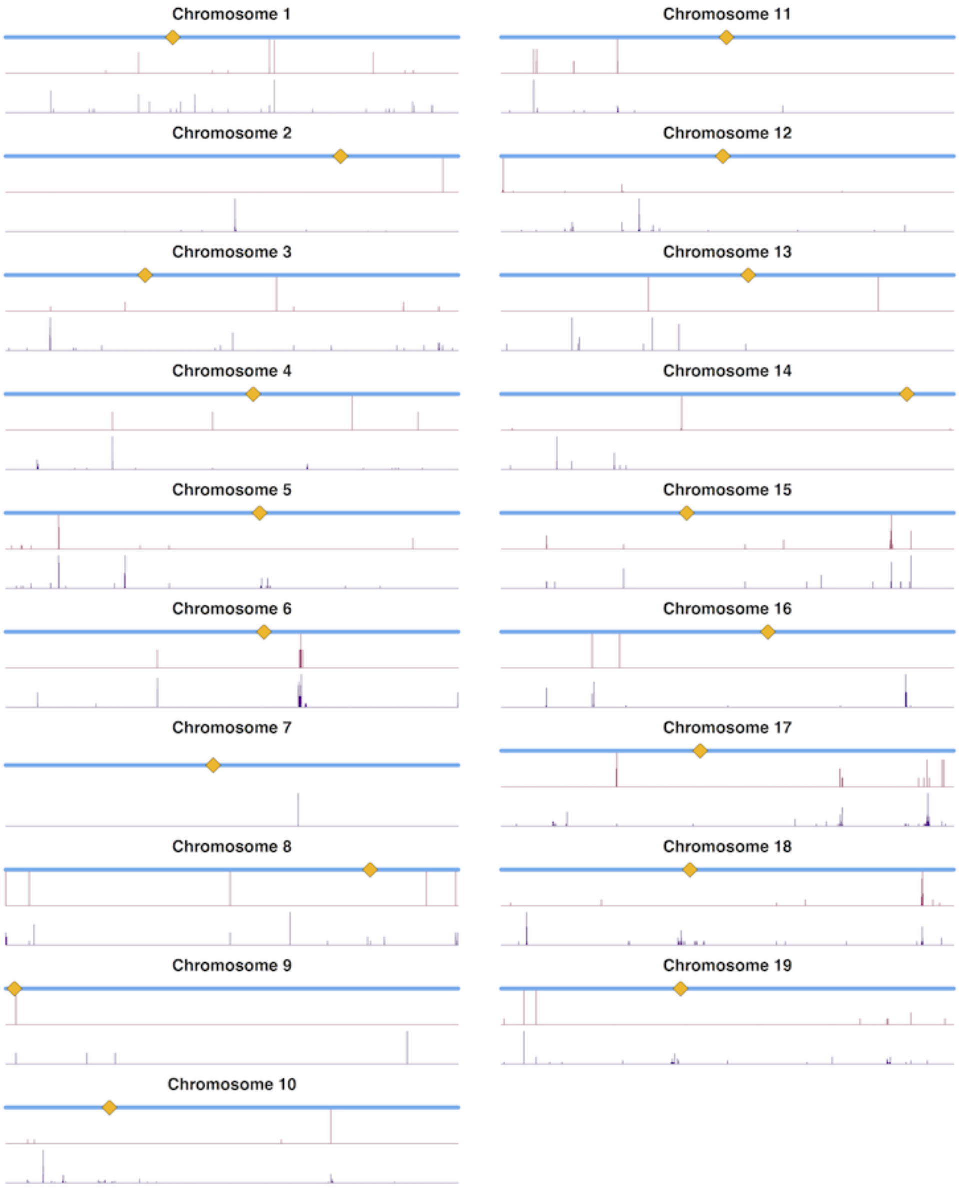

Figure S38: CENH3 gene correlations. Centromere positions (yellow diamonds) determined from wavelet coefficients and the density of SNPs correlating with SNPs in *P. trichocarpa* CENH3 genes. Red tracks are SNPs which correlate with SNPs in Potri.014G096400, and purple tracks are SNPs which correlate with SNPs in Potri.002G169000.

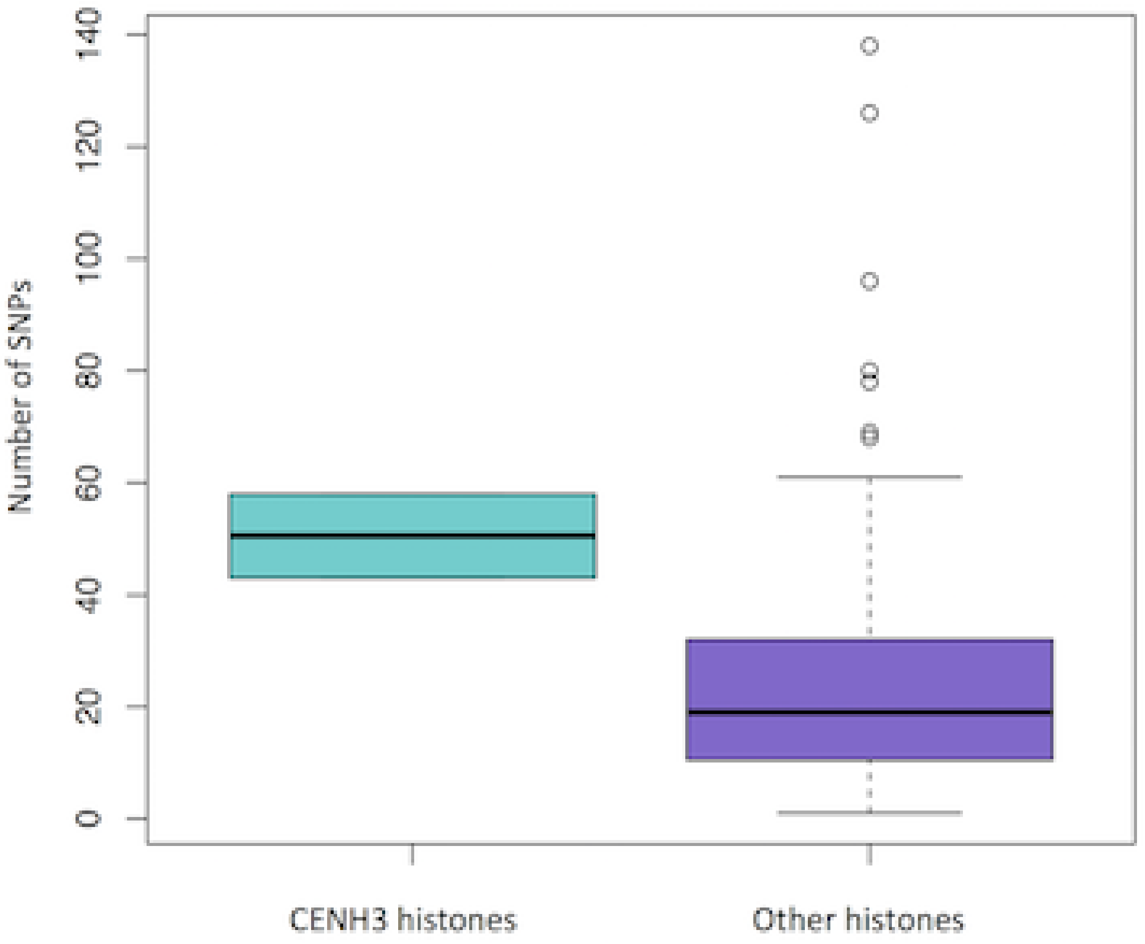

Figure S39: SNPs in *P. trichocarpa* histones. Boxplot showing the number of SNPs in the two candidate CENH3 histones versus other histones in *P. trichocarpa*.

SUPPLEMENTARY TABLES

Table S1. Positions in density signals of the approximate centromere locations, the left and right borders indicated in Figure 8 and approximate centromeric/pericentromeric length. Bp ranges indicate the borders of the particular bin in the density signal.

Chrom	Left border bin (bp)	Center bin (bp)	Right border bin (bp)	Length (Mbp)
1	12,550,001-12,560,000	18,640,001-18,650,000	24,930,001-24,940,000	12.39
2	16,680,001-16,690,000	18,690,001-18,700,000	21,170,001-21,180,000	4.50
3	4,380,001-4,390,000	6,720,001-6,730,000	7,300,001-7,310,000	2.93
4	11,770,001-11,780,000	13,270,001-13,280,000	14,710,001-14,720,000	2.95
5	12,960,001-12,970,000	14,530,001-14,540,000	16,230,001-16,240,000	3.28
6	12,510,001-12,520,000	15,930,001-15,940,000	19,270,001-19,280,000	6.77
7	5,620,001-5,630,000	7,160,001-7,170,000	8,620,001-8,630,000	3.01
8	14,670,001-14,680,000	15,670,001-15,680,000	16,790,001-16,800,000	2.13
9	1-10,000	250,001-260,000	1,610,001-1,620,000	1.62
10	4,220,001-4,230,000	5,180,001-5,190,000	6,220,001-6,230,000	2.01
11	8,390,001-8,400,000	9,210,001-9,220,000	10,770,001-10,780,000	2.39
12	6,370,001-6,380,000	7,720,001-7,730,000	9,320,001-9,330,000	2.96
13	8,400,001-8,410,000	8,910,001-8,920,000	9,500,001-9,510,000	1.11
14	14,290,001-14,300,000	16,960,001-16,970,000	18,920,001-18,930,000	4.64
15	4,480,001-4,490,000	6,260,001-6,270,000	7,980,001-7,990,000	3.51
16	7,470,001-7,480,000	8,540,001-8,550,000	9,470,001-9,480,000	2.01
17	5,700,001-5,710,000	7,070,001-7,080,000	8,980,001-8,990,000	3.29
18	6,130,001-6,140,000	7,070,001-7,080,000	8,110,001-8,120,000	1.99
19	4,970,001-4,980,000	6,320,001-6,330,000	10,440,001-10,450,000	5.48

Table S2. Pairs of SNPs passing the SNP correlation threshold of 0.7 for which one of the SNPs resides within Potri.002G169000, the putative CENH3 on *P. trichocarpa chromosome 2.*.

See attached excel sheet

Table S3. Pairs of SNPs passing the SNP correlation threshold of 0.7 for which one of the SNPs resides within Potri.014G096400, the putative CENH3 on *P. trichocarpa* chromosome 14.

See attached excel sheet

Table S4. Results of BLASTing *Arabidopsis thaliana* CENH3 against the *P. trichocarpa* genome .

See attached excel sheet

Table S5. Number of SNPs in *P. trichocarpa* histone genes.

See attached excel sheet

Table S6. Genes co-expressing with Potri.002G169000 on PhytoMine Kalderimis et al. (2014) from Phytozome Goodstein et al. (2012).

See attached excel sheet

Table S7. Genes co-expressing with Potri.014G096400 on PhytoMine Kalderimis et al. (2014) from Phytozome Goodstein et al. (2012). Blue highlighted genes are histone-related.

See attached excel sheet

REFERENCES

Goodstein, D. M., Shu, S., Howson, R., Neupane, R., Hayes, R. D., Fazo, J., et al. (2012). Phytozome: a comparative platform for green plant genomics. *Nucleic Acids Research* 40, D1178–D1186

Kalderimis, A., Lyne, R., Butano, D., Contrino, S., Lyne, M., Heimbach, J., et al. (2014). Intermine: extensive web services for modern biology. *Nucleic acids research* 42, W468–W472

Skinner, M. E., Uzilov, A. V., Stein, L. D., Mungall, C. J., and Holmes, I. H. (2009). JBrowse: A next-generation genome browser. *Genome Research* 19, 1630–1638
